# Reusable multicriteria decision model to evaluate the integrated sustainability impacts of different alternatives of dietary substitutions

**DOI:** 10.1371/journal.pone.0339454

**Published:** 2026-02-25

**Authors:** Sara M. Pires, João Fiéis de Melo, Hernan Gomez Redondo, Ricardo Assunção, Géraldine Boué, Beatrice Biasini, Elena Cozzi, Olivier Jolliet, Davide Menozzi, Ana C.L. Vieira

**Affiliations:** 1 National Food Institute, Technical University of Denmark, Lyngby, Denmark; 2 CEGIST, Instituto Superior Técnico, Universidade de Lisboa, Lisboa, Portugal; 3 Food and Nutrition Department, National Institute of Health Dr. Ricardo Jorge, Av. Padre Cruz, Lisboa, Portugal; 4 Oniris, INRAE, SECALIM, Nantes, France; 5 Department of Food and Drug, University of Parma, Parco Area delle Scienze, Parma, Italy; 6 Department of Environmental and Resource Engineering, Technical University of Denmark, Lyngby, Denmark; PLOS, UNITED KINGDOM OF GREAT BRITAIN AND NORTHERN IRELAND

## Abstract

Policies promoting shifts towards sustainable diets must consider the health, environmental, and socioeconomic impacts of changes and the trade-offs among these impacts across options. Comprehensive assessments are challenged by data uncertainties, diverse metrics, and conflicting interests of stakeholders. The aim of this study was to build a reusable multicriteria model to evaluate the integrated impacts of food substitutions. We applied a multicriteria decision analysis (MCDA) approach that combined input from eight multi-disciplinary experts with evidence collected from scientific literature and public databases across four dimensions: health, environment, economics and social implications of food systems. We tested the approach by assessing the impact of substituting beef with equivalent amounts of pulses in the Portuguese and Danish diets in four scenarios. Results demonstrated an overall positive impact of replacing beef with pulses in both populations, with benefits increasing incrementally with greater levels of substitution. This multicriteria model is adaptable to other contexts and populations, thereby assisting in the development of food policies that consider both health and sustainability concerns.

## 1. Introduction

The recognized negative impacts of current food systems on the health of populations and the environment call for urgent actions to the way that food is produced, distributed, and consumed. Shifting towards sustainable and healthier diets, such as substituting meat with proteins from alternative sources, can mitigate these impacts [1,2]. Meat production is a major driver of environmental degradation, consuming key resources like water and land, and contributing to biodiversity loss [3]. High consumption of red meat is also a leading health risk [4–6]. Recently, alternative proteins like plant-based options, cell-based meat, edible insects, and microalgae have gained popularity [7].

Transforming food systems through dietary change involves navigating multiple, often complex objectives: improving health, reducing environmental impacts, enhancing economic outcomes, and ensuring social acceptability [8]. No single, universally applicable solution exists to drive shifts in consumption patterns toward sustainability and health. This transition requires a systems-based approach that combines diverse strategies to support informed consumer choices and improve efficiency across the food supply chain.

Despite this complexity, much of the existing research on dietary substitutions remains siloed, addressing impacts within individual domains [9–15]. While domain-specific assessments are valuable, they may overlook the synergies and trade-offs across health, environmental, economic, and social dimensions that are essential for policy relevance.

Comprehensive evaluation of dietary alternatives across these dimensions is critical to designing effective food and public health policies. This calls for integrated analytical frameworks that explicitly identify co-benefits and transparently characterize trade-offs, enabling policymakers to make balanced decisions. Interdisciplinary collaboration is essential to develop such frameworks and ensure their relevance in real-world decision-making contexts [16].

Integrated assessments of dietary shifts are hampered by gaps in data availability and completeness, which introduce substantial uncertainty into impact estimates. Moreover, the use of heterogeneous metrics across domains further complicates synthesis: health outcomes may be expressed as changes in disease incidence, mortality, or disability‑adjusted life years [17]; economic impacts as changes in gross value added, employment, consumer food expenditure, or price volatility [9,18]; and environmental impacts as greenhouse‑gas emissions, alterations in air, soil, and water quality, or rates of land degradation [9,19]. Various studies have attempted multidimensional integration, applying for example optimization modelling [20,21], radar plots [22], coefficients of association [23], and regression models [15,24]. However, these efforts frequently omit key elements such as biodiversity impacts, broader economic externalities, or measures of consumer acceptance, thereby limiting their scope and precluding truly holistic appraisals. To advance policy‐relevant research, future frameworks must reconcile domain‑specific indicators within a unified architecture, fill critical data gaps, and incorporate under‑represented dimensions to capture the full spectrum of co‑benefits and trade‑offs.

Multiple Criteria Decision Analysis (MCDA) provides structured methods to support decision-makers when faced with multiple, often conflicting objectives. As an umbrella term, MCDA encompasses methods that systematically evaluate trade‑offs and inform high‑stakes policy choices [25]. For instance, Agyemang et al. (2022) applied an MCDA protocol to assess food‑system interventions across health, environmental, economic, and social sustainability dimensions, illustrating how multicriteria tools can shape more coherent and evidence‑based policy design [26]. However, the approach applied by Agyemang and colleagues relies on ordinal rankings alone and does not quantify the intensity of stakeholder preferences across criteria. Although it identifies which options lie closest to an ideal reference scenario, it fails to account for how much more (or less) desirable one alternative is relative to another or the magnitude of their divergence. Consequently, two strategies may occupy adjacent positions in the ranking while differing substantially in feasibility, resource requirements, or expected impact—an ambiguity that can mislead policymakers. To strengthen food-system decision-making, MCDA applications must go beyond simple ranking and integrate preference elicitation techniques.

MCDA methods grounded in value measurement directly address these gaps by quantifying the strength of decision-maker preferences. An example is MACBETH (Measuring Attractiveness by a Categorical Based Evaluation Technique), which constructs a numerical value model from qualitative pairwise judgments — “null,” “weak,” “moderate,” or “strong”—to capture value differences between options [27]. By asking stakeholders not only which alternative they prefer but by how much, MACBETH provides nuanced insights into trade‑off intensity rather than mere ordinal rankings. This feature is especially pertinent for food‑system decisions, where trade‑offs can range from marginal (e.g., slightly higher consumer prices) to profound (e.g., job losses in livestock sectors). For example, replacing animal‑based products with plant‑based alternatives may yield substantial environmental and health gains, yet incur economic and social costs that vary in stakeholder salience. MACBETH’s structured pairwise comparisons reduce cognitive burden while preserving flexibility, enabling decision‑makers to articulate fine‑grained value judgments and distinguish minor concessions from critical conflicts [27–30]. MACBETH has been applied across domains, including health [27,29–31]. The outcome of a value measurement approach can support policymakers develop solutions that are not only technically optimal but also context-sensitive and publicly acceptable, ensuring more effective and equitable transitions toward sustainable food systems [32–34].

The aim of this study was to build a reusable multicriteria model aligned with value measurement principles to evaluate the integrated impacts of different alternatives of dietary changes [34]. Specifically, the goal was to gather and combine expertise in the health, environmental, economic and social domains to develop and implement an approach for the evaluation of the integrated impact of replacing animal-based protein sources with more sustainable alternatives in a population. The model was tested by evaluating four simplified scenarios of replacement of beef consumption by equivalent amounts of pulses in two countries, Portugal and Denmark. We engaged a multidisciplinary group of eight experts combining disciplinary diversity (health, environment, economics, social sciences) with cultural and national diversity (Portugal, Denmark, France, Italy), ensuring a broad representation of perspectives on European food systems, and combined structured expert elicitation with evidence synthesis and applying a value-measurement approach. This study was developed within the scope of the project ALTERNATIVA (“Alternative protein sources in the European diets – integrating health risk-benefit and sustainability”) [35].

## 2. Methods

### 2.1. Scope

We investigated the overall impact of different alternatives of substitution of unprocessed beef with pulses in Denmark and Portugal, considering health, social, economic and environmental impacts of these substitution in each country. The two food categories represent an animal and a plant-based protein source that are well-established in European diets. The two countries were selected because they represent distinct dietary patterns, including sources of proteins [36], gastronomic culture, geography, and socioeconomic setting.

### 2.2. Overview of the socio-technical approach

**Fig 1:**
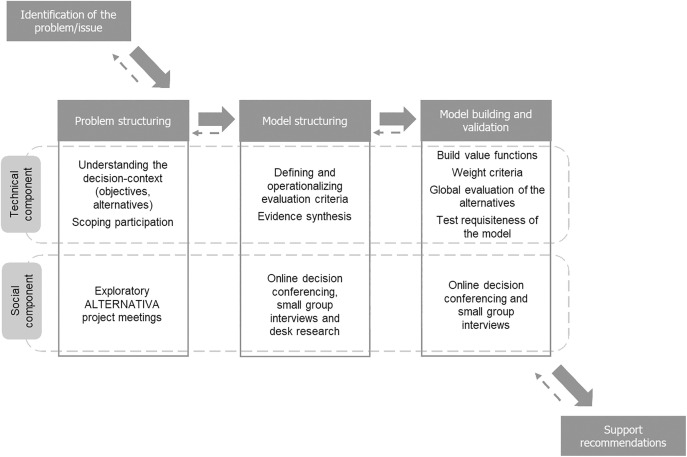
Socio-technical approach followed for developing the reusable multicriteria evaluation model to assess the integrated health and sustainability impacts of specific dietary changes in populations (adapted from [40,37].

We structured our approach into three distinct phases: problem structuring; model structuring; and model building and validation, described in detail in the next sub-sections and illustrated in Fig 1. The three phases were carried out between January and May of 2023 in an iterative sequence, which enabled those involved in the decision-making process to revisit previous phases and adjust as needed, aligning with the constructivist perspective commonly employed multicriteria value measurement [37]. Based on the overall impact of the difference in assessment, we produced recommendations. The socio-technical approach described was implemented with the support of a facilitation team with experience in problem structuring methods and MCDA methods and support tools. The participatory context in which the study was conducted was aligned with decision conferencing principles, which emphasize the interactive, iterative development of multicriteria value models within a focused group of key stakeholders [33]. Decision conferencing has been widely used for complex decision-making processes, particularly in MCDA, as it enables in-depth deliberation, conflict resolution, and consensus-building among experts [38,39]. To capture diverse perspectives and align them with the decision-making objectives, frame the problem accurately, and guide the analysis towards relevant and actionable insights, we selected experts with expertise in at least one of the relevant areas, particularly in the context of food systems and diets, to participate in all steps of the multicriteria model building process. We initially selected experts from the multi-disciplinary consortium of the ALTERNATIVA project [35], which comprised partners from six academic and governmental institutions in four countries (Portugal, France, Italy, Denmark). Four dimensions were determined relevant: social; economic; health; environmental, which constitute main pillars of sustainability. We selected experts based on pre-defined criteria, namely: i) scientific expertise in one or more of the dimensions relevant to the study, with a focus on food systems (i.e., nutrition and public health; environmental impacts of food systems; agrifood economics; consumer perceptions and attitudes); ii) active roles in research, risk–benefit assessment, or sustainability evaluation; and iii) institutional affiliation that could contribute to balanced representation of perspectives. To ensure a balanced representation of the dimensions by the project members, we invited an additional (external) expert within the environmental domain. The final group was constituted of eight participants, which collectively, covered nutrition, microbiology, toxicology, epidemiology, agrifood economics, consumer behaviour, and environmental sciences, thereby providing a qualified panel consistent with the objectives of the ALTERNATIVA project. This core group was actively involved in all stages of the study, including problem structuring, criteria definition, evidence review, and preference elicitation during decision-conferencing sessions. Their expertise directly informed the development and validation of the decision model. All participants provided verbal consent for their responses to be recorded and used in the analysis. This consent was documented in meeting minutes. Ethical approval from an Institutional Review Board (IRB) was not required, as the study did not involve medical or psychological interventions, did not collect sensitive personal data, and was conducted as part of an internal project decision-making process. Participation was voluntary, no personal information was collected, and all data were anonymized prior to analysis.

### 2.3. Problem structuring

We held three exploratory online meetings over the course of one month with the key experts identified from the ALTERNATIVA project. These meetings were crucial for understanding the complex issues at hand, such as the diversity – and possibly divergence – of environmental, health, economic, and societal impacts of substituting animal-based proteins with sustainable alternatives. During these discussions, it was also established that there was value in developing an integrated assessment approach that can be reused to assess the impacts of different possible dietary substitutions, and thus that the evaluation model would need to accommodate the possibility to compare different alternative protein sources, considering that there are several animal-based protein sources in current diets as well as multiple protein alternatives. This decision had implications in the next phase of model structuring.

These exploratory meetings were also essential for selecting which alternatives were going to be selected to test the reusable evaluation model. The group decided that the model would be tested by answering the question: “What is the integrated impact of replacing different amounts of beef consumption by equivalent amounts of pulses in Denmark and in Portugal?” We chose four potential substitution scenarios of replacement of beef with equivalent amounts of pulses (in grams per day) in each country: replacing 25% (Alternative Scenario (AS) 1); 50% (AS2); 75% (AS3); or 100% (AS4). The scenarios were selected to represent potential gradual increases of replacement of beef with pulses. A reference scenario captured the current consumption of both foods in Portugal and Denmark. From this baseline, four hypothetical substitution scenarios were constructed, in which 25%, 50%, 75% and 100% of beef consumption was replaced while holding other dietary components constant. This design provided transparent, comparable counterfactuals across countries and comprises a range of plausible substitution magnitudes, from incremental to full replacement. It also allowed the MCDA to analyse the impact of each scenario by criterion and country.

### 2.4. Model structuring

The model structuring phase included the definition of evaluation criteria (i.e., independent evaluation axes upon which the alternatives are evaluated) and the construction of descriptors of performance for operationalizing the criteria. To define the evaluation criteria, the facilitation team guided the group of experts towards the development of a value tree, i.e., a simple visual and structured representation that helps conceptualize and structure the problem [33]. To identify the evaluation criteria for the model, we adapted the “post-it sessions” method, where participants write relevant concerns on post-its and group similar issues on a board [25], to a virtual setting using the platform Ideaboardz. A videoconference of two hours was held on January 12th, 2023. All experts from the four dimensions joined. Ideaboardz was divided into sections for each dimension of the problem (health, environmental, economic, and social) to prompt focused thinking. Participants voted for the concerns they agreed with, followed by an open discussion session where concerns were grouped, selected, and refined until they met the key criteria proprieties [41]. Although each expert brought primary expertise in one dimension, the process encouraged all participants to comment across domains, consistent with the constructive perspective of the study and aiming at group agreement. An executive summary containing the list of criteria and their detailed description was circulated post-meeting for participant reflection. An online spreadsheet was also provided for further suggestions, with a two-week deadline for validation. Based on feedback, some criteria were added, removed, or modified. The final value tree was validated by all participants.

We then moved on to constructing the descriptors of performance – an ordered set of plausible performance levels associated with each specific criterion [41]. The goal is to describe each alternative’s performance on a criterion as objectively as possible. This step was divided by dimension of the problem (health, environmental, economic, and social), with six decision conferences held over six weeks with the relevant representatives for each dimension. We used the M-MACBETH software for this process [42]. In these meetings, we defined the descriptors for each criterion with the respective reference performance levels (neutral and good levels of performance that described which was a acceptable level of performance and a good one, respectively, for each group of experts), and evaluated each alternative for the two food systems in the two countries by attributing a level of performance in each criterion. For the latter, we collected and synthesized evidence (performance data) from open-access databases and the scientific literature, restricted to the period between 2003 and 2023. When performance data on the alternatives were lacking for one of the food systems or country, we collected the raw data required to estimate the performance information. The data sources and detailed estimates for specific indicators are presented in S3 Appendix. All collected performance data were synthesized and presented to the experts for review.

### 2.5. Model building and validation

During model building, we conducted two main activities: building value scales, so that the performance of each scenario on the criteria was converted into a partial value score, and assigning criteria weights, to be able to transform the partial value scores into global value scores. The global value scores of the alternatives were then calculated as follows:


$$V(a)=\;\sum {\mathrm{\backslash nolimits}}_{j=\text{1}}^{n}w_{j}v_{j}(a),\;\;with\;\sum {\mathrm{\backslash nolimits}}_{j=\text{1}}^{n}w_{j}=\text{1},\;\;w_{j}>\text{0}\;$$ (1)

where V(a) represents the global value of an alternative a, v_j (a) translates the partial value of the alternative a in criterion j, and w_j is the weight of criterion j. In each criterion, the neutral (minimally acceptable) and the good performance levels (see Table 1) were associated to 0 and 100 points, respectively.

**Table 1:** Overview of the environmental, social, economic, and health criteria, with the identification of the rational for inclusion in the model, the respective descriptor of performance and cardinal value scale, and data source of collected evidence used to assess the impacts of dietary substitutions.

Criteria	Rational	Descriptor of performance	Data source	Cardinal value scale
Profitability across the supply chain: financial performance of the farms	Profitability of all production processes across the supply chain, measured as the ration of gross margin and revenue	L1: 60 L2: 40 (Good) L3: 20 L4: 0 (Neutral) L5: −20 L6: −40 L7: −60	Estimated from [45–50]	L1: 150 L2: 100 L3: 50 L4: 0 L5: −50 L6: −108.33 L7: −166.67
Affordability for consumers	Affordability of the end product is for consumers in the population, measured in Purchasing Power Parity	L1: 60 L2: 30 L3: 10 (Good) L4: 0 (Neutral) L5: −10 L6: −30 L7: −60	FAOSTAT database – year 2017 and *GlobalProductPrices.Com,* year 2023 [51]	L1: 300 L2: 200 L3: 100 L4: 0 L5: −100 L6: −200 L7: −333.3
Local economic development	Impacts to local economies, based on employment and economic spill-over on the territory	L1: More employment, local multiplier >2 (Good) L2: No change in employment, local multiplier >2 L3: More employment, local multiplier <2 L4: No change in employment, local multiplier <2 (Neutral) L5: Loss of employment, local multiplier >2 L6: Loss of employment, local multiplier <2	[52], Eurostat database, year 2016 [53]	L1: 100 L2: 66.67 L3: 33.33 L4: 0 L5: −33.33 L6: 66.67
Impact on local communities: Local economic development	Impacts to local economies, based on employment and economic spill-over on the territory	L1: More employment, local multiplier >2 (Good) L2: No change in employment, local multiplier >2 L3: More employment, local multiplier <2 L4: No change in employment, local multiplier <2 (Neutral) L5: Loss of employment, local multiplier >2 L6: Loss of employment, local multiplier <2	[53], Eurostat database, year 2016 [53]	L1: 100 L2: 66.67 L3: 33.33 L4: 0 L5: −33.33 L6: 66.67
Acceptance of the change	How much people are willing to change their dietary habits and if there are food-related misconceptions that may have an impact	L1: Willingness to change, no food misconceptions (Good) L2: Willingness to change, food misconceptions exist (Neutral) L3: No willingness to change, no food misconceptions L4: No willingness to change, food misconceptions exist	Estimated in the MCDA sessions	L1: 100 L2: 0 L3: −133.33 L4: −233.33
Fair and ethical practices: Female labour force	Contribution of the food’s value chain to employment of women in the population	L1: Higher % of women employed, higher % of women in management L2: Higher % of women employed, similar % of women in management (Good) L3: Higher % of women employed, lower % of women in management L4: Similar % of women employed, similar % of women in management L5: Similar % of women employed, lower % of women in management (Neutral) L6: Lower % of women employed, higher % of women in management L7: Lower % of women employed, similar % of women in management L8: Lower % of women employed, similar % of women in management L9: Lower % of women employed, lower % of women in management	Eurostat database, year 2016 [53]	L1: 127.27 L2: 100 L3: 72.73 L4: 45.45 L5: 0 L6: −27.27 L7: −54.55 L8: −127.27 L9: −145.45
Accessibility	Ease of access to the foods	L1: More accessible (Good) L2: As accessible (Neutral) L3: Less accessible	Estimated in the MCDA sessions	L1: 100 L2: 0 L3: −133.33
Diet-related health impacts	Impact on the population’s health through changes in burden of disease associated with dietary risk factors. Measured in DALYs*/100,000 inhabitants	L1: −400 L2: −300 L3: −200 L4: −100 (Good) L5: 0 (Neutral) L6: 100 L7: 200 L8: 300 L9: 400	[54,55] Calculated based on [56]	L1: 400 L2: 300 L3: 200 L4: 100 L5: 0 L6: −100 L7: −200 L8: −300 L9: −400
Environmental-related health impacts	Impact on the population’s health, through the environmental impacts of the food system	L1: 0 L2: 0.1 (Good) L3: 0.3 (neutral) L4: 0.5 L5: 1 L6: 2 L7: 3		L1: 150 L2: 100 L3: 0 L4: −100 L5: −200 L6: −300 L7: −400
Biodiversity impact	Impact of the food’s production on biodiversity (measured in number of species destined to extinction per year)	L1: 0 (Good) L2: 5 L3: 15 L4: 28 (Neutral) L5: 50 L6: 100 L7: 250	[57]	L1: 100 L2: 66.67 L3: 33.33 L4: 0 L5: −83.33 L6: −200 L7: −283.33
Climate change	Contribution of the food products´ production process to GHG emissions (measured in GHG emissions per kg of food [kg CO_2_ eq])	L1: 0 L2: 0.3 (Good) L3: 0.6 (Neutral) L4: 1.5 L5: 4	[57]	L1: 133.33 L2: 100 L3: 0 L4: −166.67 L5: −333.33
Water use	Impact of the production process on the use of water resources (measured in Water use per kg of food [L/Kg])	L1: 0 L2: 5 L3: 10 (Good) L4: 30 L5: 50 L6: 70 L7: 90 (Neutral) L8: 200 L9: 350 L10: 560	[57]	L1: 130 L2: 115 L3: 100 L4: 70 L5: 40 L6: 20 L7: 0 L8: −40 L9: −80 L10: −120
Land use	Impact of the production process on the use of land resources (measured in arable and pasture use per kg of food)	L1: 0 L2: 1.5 (Good) L3: 3 (Neutral) L4: 5 L5: 10 L6: 20 L7: 40	[57].	L1: 200 L2: 100 L3: 0 L4: −100 L5: −300 L6: −500 L7: −900
Pollution: Water Eutrophication	Impact of the use of fertilisers and combustion processes on excessive plant and algal growth (measured in g of phosphate per serving size)	L1: 0 L2: 1 (Good) L3: 5 (Neutral) L4: 10 L5: 25 L6: 40	[57]	L1: 150 L2: 100 L3: 0 L4: −100 L5: −300 L6: −500

*DALY: Disability-adjusted life years

For the model developed in this study, the global value of each alternative was obtained by applying this formula to the 14 criteria included in the final value tree (Fig 3). The resulting equation was:

**Fig 2:**
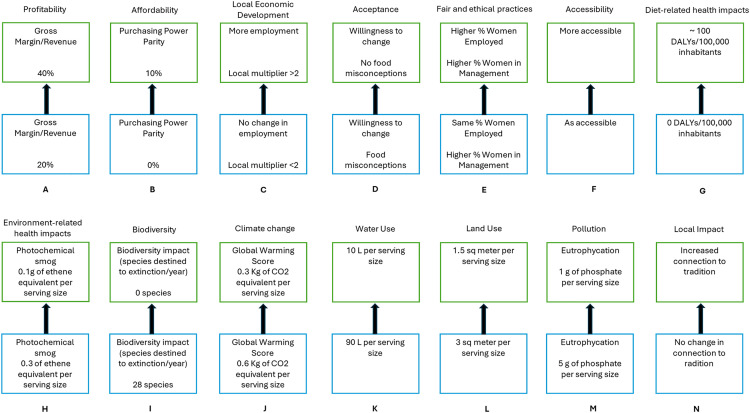
Visual support for the swing weighting task, depicting the 14 criteria (A-N) and respective swings between Neutral (blue) and Good (green).


$$\begin{array}{cc} \text{V}(\text{a})= & \;\text{0}.\text{0605}\cdot \text{vProfitability}(\text{a})+\text{0}.\text{0968}\cdot \text{vAffordability}(\text{a})+\text{0}.\text{0403}\cdot \text{vLocal}\;\text{development}(\text{a})\\ & +\text{0}.\text{0081}\cdot \text{vImpact}\;\text{on}\;\text{local}\;\text{communities}(\text{a})+\text{0}.\text{0524}\cdot \text{vAcceptability}(\text{a})\\ & +\text{0}.\text{0282}\cdot \text{vFair},\text{ethical}\;\text{practices}(\text{a})+\text{0}.\text{0202}\cdot \text{vAccessibility}(\text{a})\\ & +\text{0}.\text{1210}\cdot \text{vDiet}-\text{related}\;\text{health}\;\text{impacts}(\text{a})+\text{0}.\text{0766}\cdot \text{vEnvironment}-\text{related}\;\text{health}\;\text{impacts}(\text{a})\\ & +\text{0}.\text{1129}\cdot \text{vBiodiversity}(\text{a})+\text{0}.\text{1250}\cdot \text{vClimate}\;\text{change}(\text{a})+\text{0}.\text{1048}\cdot \text{vWater}\;\text{use}(\text{a})\\ & +\text{0}.\text{0685}\cdot \text{vLand}\;\text{use}(\text{a})+\text{0}.\text{0847}\cdot \text{vPollution}(\text{a})\;\;\;\;\; \end{array}$$ (2)

where vj(a) denotes the partial value of alternative a on criterion j, and *wj* denotes the weight of criterion *j* as elicited from the expert panel. All weights are strictly positive and normalized to sum to 1 (see Table 4).

We used MACBETH to build the value scales and assign the weights. Both model building activities were performed within (online) decision conferences [43] held with the experts and aided by M-MACBETH and StrawPoll (https://strawpoll.com/) decision support systems. The value scales within each dimension were built within smaller groups, generally the same groups that performed the criteria operationalization tasks, that had expertise in that specific domain. Using the reference levels that were established for each descriptor of performance, the participants were required to judge the differences in attractiveness between reference levels, by performing pairwise comparisons through the use of MACBETH semantic categories: “null”, “very weak”, ”weak”, “moderate”, “strong”, ”very strong” and “extreme”. The questioning protocol to elicit judgements consisted of questions such as ”When evaluating alternatives, what is the difference in attractiveness between an alternative with [Level 1] and [Level 4]?”, where ”Level 1” and ”Level 4” represented levels of the criterion’s descriptor of performance.” The judgements were first collected individually within each subgroup using StrawPoll, then discussed to agree on the final entries that were compiled into a M-MACBETH judgement matrix. The software then employs an algorithm to originate value scales from the judgements entered in the matrix, which will allow the conversion of performance into (partial) value [44]. All resulting value scales were then validated by the participants, with questions such as “Regarding criterion X, do you agree that an improvement from level L4 to level L3 is twice as attractive as an improvement from level L3 to level L2?”.

According to the MACBETH method, there are three technical tasks that the participants must complete to assign weights to the criteria. First, the criteria were ranked according to the attractiveness of the swing from the”Neutral” to the”Good” reference levels; second, the M-MACBETH weighting matrix was filled by qualitatively judging each swing (i.e., the comparison of the attractiveness of moving from a neutral level of performance towards a good level of performance); third, participants were asked to evaluate the differences in attractiveness between the swings, by performing pairwise comparisons between them using the semantic scale from the M-MACBETH software. Fig 2 shows the visual support for the swing weighting task. Much like the process used to build the value scales, the judgements were used to build a matrix, which was checked for inconsistencies in real-time. Once the matrix was consistent and validated by the participants, the software calculated coefficient weights for each criterion and generated a histogram, that helped the participants perform the necessary validation. Unlike the criteria operationalization and value scale construction, the weighting exercises were performed with the entire group of stakeholders within a separate online decision conferencing session that took place on April 27^th^, 2023.

Model testing and validation was performed during a final online decision conference where the group of experts discussed the model’s results and performed, assisted by the facilitator, sensitivity and robustness analyses. The sensitivity analysis was done to evaluate how changes in some criteria weights would affect the outcome of the model, by analysing how much a certain criterion weight would need to change to have an impact on the final recommendation. The robustness analysis was done to evaluate the participants’ judgements, by adding uncertainty to their choices and analysing the dominances between two alternatives at a time. Once these two analyses were completed, there was sufficient information to relay a final recommendation.

## 3. Results

### 3.1. Model structuring

**Fig 3:**
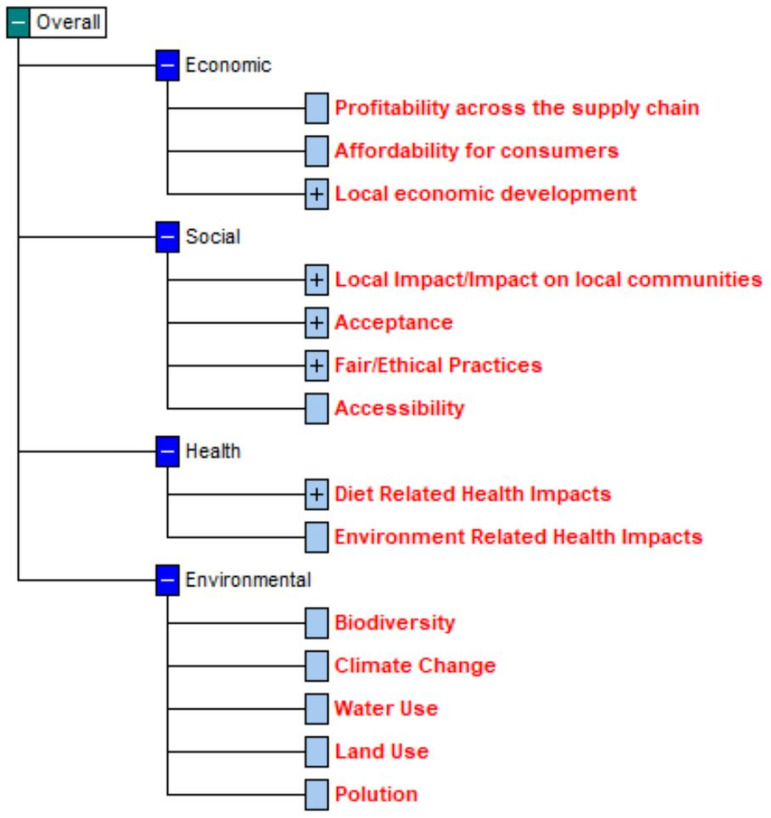
Value tree that depicts the four dimensions, with the respective criteria highlighted in bold red identified to assess the integrated health and sustainability impacts of different scenarios of dietary substitutions.

The experts identified 14 criteria to assess alternatives, which were organized into four dimensions. The value tree that represents the decision problem, with the criteria highlighted in bold red, is presented in Fig 3. In S1 Appendix we detail the process of defining the criteria, from the “post-it sessions” to the final validation by the group of experts.

On Table 1 we present the descriptors of performance for the 14 criteria (with different levels of performance, L, and the Neutral and Good reference levels identified). The list and description of the final criteria are presented in S2 Appendix. The evidence gathered to assess the 14 indicators in the two countries is presented in Tables 2 and 3. In Table 1, we also report the cardinal scales obtained with the M-MACBETH software for each descriptor of performance.

**Table 2:** Evidence synthesis of the impacts of environmental, health, social and economic indicators in four scenarios of substitution of consumption of beef by pulses in Denmark.

Indicator	Reference Scenario	Alternative Scenario 1 (25% Substitution)	Alternative Scenario 2 (50% Substitution)	Alternative Scenario 3 (75% Substitution)	Alternative Scenario 4 (100% Substitution)
**Environmental dimension**					
GHG emissions (kg CO2eq)	1.2	0.9	0.6	0.4	0.1
Eutrophication (g PO43- eq)	4	3.1	2.2	1.3	0.5
Water Use (L)	33.5	29.5	25.5	21.4	17.4
Arable Land Use (m2*year)	0.8	0.7	0.6	0.5	0.4
Pasture Land Use (m2*year)	0.3	0.2	0.1	0.1	0
Total Land Use (m2*year)	1	0.9	0.7	0.5	0.4
Biodiversity impact	54.4	42.4	30.5	18.5	6.6
**Health dimension**					
Colorectal cancer (DALYs)		−5.4 (0; −23.)	−10.7 (0; −46.7)	−15.9 (0; −68.4)	−20.9 (0; −89.2)
Diabetes (DALYs)		−1.1 (0; −5.4)	−2.1 (0; −10.6)	−3.1 (0; −15.5)	−4.1 (0; −20.1)
Ischemic heart disease (DALYs)		−4.1 (0; 17.3)	−7.9 (0; −32.4)	−11.5 (0; −45.5)	−14.7 (0; −56.8)
Total DALYs		−10.7 (0; −46.6)	−20.8 (0; −89.6)	−30.5 (0; −129.4)	−39.7 (0; −166.2)
**Social dimension**					
Female manager/holder [AWU]	520	882	1243	1605	1966
Farm labour Female [AWU]	1570	2678	3786	4894	6002
Female Farm managers, excluding group holding [AWU]	280	456	632	807	983
Affodability [PPP]	1.1	0.85	0.61	0.36	0.12
Economic dimension*					
GOM as % of turnover (relative change in % vs. baseline)		−7,90	−15,79	−23,69	−31,59
Employment (relative change in % vs. baseline)		−22,26	−44,53	−66,79	−89,05
Local multiplier (relative change in % vs. baseline)		0,62	1,25	1,87	2,50

GHG: Greenhouse gas. DALYs: Disability adjusted life years. AWU: annual working unit. PPP: purchasing power parity. GOM: gross operating margin. *Data should be interpreted as % of variation compared to the baseline (status quo) across the different scenarios. For example, in Denmark the 100% substitution of beef with pulses (AS4) might result in a reduction of 32% in gross operating margin, −89% drop in regional workforce (employment), and an increase in the local spill-over (the effect of one euro spent in the downstream supply chain within the local economy) by 2.5%.

**Table 3:** Evidence synthesis of the impacts of environmental, health, social and economic indicators in four scenarios of substitution of consumption of beef by pulses in Portugal.

Indicator	Reference Scenario	Alternative Scenario 1 (25% Substitution)	Alternative Scenario 2 (50% Substitution)	Alternative Scenario 3 (75% Substitution)	Alternative Scenario 4 (100% Substitution)
**Environmental dimension**					
GHG emissions (kg CO2eq)	0.8	0.6	0.4	0.2	0
Eutrophication (g PO43- eq)	4.5	3.5	2.4	1.4	0.3
Water Use (L)	19.9	17.8	15.8	13.7	11.7
Arable Land Use (m2*year)	0.4	0.4	0.3	0.3	0.3
Pasture Land Use (m2*year)	1.9	1.4	0.9	0.5	0
Total Land Use (m2*year)	2.3	1.8	1.3	0.8	0.3
Biodiversity impact	28.6	22.6	16.5	10.5	4.4
**Health dimension**					
Colorectal cancer (DALYs)		−1.6 (0; −15.8)	−3.2 (0; −30.7)	−4.6 (0; −44:8)	−6 (0; −58.2)
Diabetes (DALYs)		−0.4 (0; −3.2)	−0.7 (0; −6.1)	−1.1 (0; −8.8)	−1.4 (0; −11.2)
Ischemic heart disease (DALYs)		−3.8 (0; −36.5)	−7.2 (0; −67.5)	−10 (0; −93.8)	−12.6 (0; −116.2)
Total DALYs		−5.9 (0; −55.5)	−11.1 (0; −104.3)	−15.8 (0; −147.4)	−20 (0; −185.6)
**Social dimension**					
Female manager/holder [AWU]	9140	9436	9732	10027	10323
Farm labour Female [AWU]	11110	11353	11596	11839	12082
Female Farm managers, excluding group holding [AWU]	3950	4236	4521	4807	5092
Affodability [PPP]	0.38	0.31	0.24	0.17	0.10
Economic dimension*					
GOM as % of turnover (relative change in % vs. baseline)		13,21	26,42	39,63	52,84
Employment (relative change in % vs. baseline)		−11,99	−23,99	−35,98	−47,97
Local multiplier (relative change in % vs. baseline)		0,62	1,25	1,87	2,50

GHG: Green house gas. DALYs: Disability adjusted life years. AWU: annual working unit. PPP: purchasing power parity. GOM: gross operating margin. *Data should be interpreted as % of variation compared to the baseline (status quo) across the different scenarios. For example, in Denmark the 100% substitution of beef with pulses (AS4) might result in a reduction of 32% in gross operating margin, −89% drop in regional workforce (employment), and an increase in the local spill-over (the effect of one euro spent in the downstream supply chain within the local economy) by 2.5%.

The measurements of performance of all criteria for each substitution scenario as evaluated by the stakeholders based on this evidence showed that, for Denmark, the profitability performance decreased with each scenario with higher substitution of beef by pulses (i.e., was higher for 0% substitution and lowest for 100% substitution). The performance measurements for Portugal were opposite, with increasing performance with each scenario. In contrast, the affordability performance increased from the scenario 0% to 100% in the two countries (see details in S4 Appendix). The performance of the criteria of the social dimension were less straightforward, or the same for each scenario (in the case of “accessibility”). The performance of the health criteria improved with each increasing scenario in both countries and was particularly marked for the diet-related health impacts, ranging from 0 (0% substitution scenario) to −39.7 (100% substitution) in Denmark, and from 0 to −20 in Portugal (see S4 Appendix). This shows that, with each increase in substitution of beef by pulses, the beneficial health impacts increased. The health benefits were more marked in Denmark. The performance of all environmental criteria was also higher with each scenario in both countries, showing that the negative environmental impacts of the food systems decreased with increasing proportions of replacement of beef consumption by pulses in the two populations. The performance of “local economic development” was the same for all alternatives in the two countries, showing that the performance level was the same for these specific scenarios. Detailed results are presented in S4 Appendix.

### 3.2. Model building

Table 4 presents the partial score for the four alternatives in each criterion in each country, each alternative’ overall score in each country (in bold), ranked from higher global score to lower global score, and the criteria weights (last row). The results indicate that the alternative “100% substitution of beef by pulses” was the most attractive in both countries, with an overall score of 102.4 in Portugal and 97.7 in Denmark, followed by the alternatives”75%”, “50%”, “25%” and “0% substitution”. The overall score increased with each increase in the percentage of substitution of beef by pulses. These overall results reflect the partial value of each alternative in each criterion in each country, considering the weights of criterion (which are independent of alternatives and country). The criteria with higher weights were”diet-related health impact”, “biodiversity”, “climate change”, and “water usage”; the ones with lower weights were “impact to local development” and “accessibility of the food” (see last line of Table 4).

**Table 4:** Overall global assessment of the four alternatives in Portugal (PT) and Denmark (DK), partial score for the four alternatives in each criterion in each country, and criteria weights (last row).

Alternatives (% Substitution)	Overall	Profitability across the supply chain	Affordability for consumers	Local economic development	Impact on local communities	Acceptability	Fair/ethical practices	Accessibility	Diet-related health impact	Environmental related health impacts	Biodiversity	Climate change	Water usage	Land Usage	Pollution
PORTUGAL															
100%, PT	**102.37**	132	346.67	−66.67	0	−233.33	127.27	0	20	145	70.67	133.33	97.45	180	135
75%, PT	**80.05**	99	286.67	−66.67	0	−233.33	127.27	0	15.9	115	48.33	111.11	94.45	146.67	90
50%, PT	**57.07**	66	223.33	−66.67	100	−233.33	127.27	0	11.1	80	29.48	66.67	91.13	113.33	65
25%, PT	**40.89**	33	140	−66.67	100	0	127.27	0	5.9	45	13.84	0	88.3	80	37.5
0%, PT	**16.99**	0	0	66.7	0	100	0	0	0	10	−2.27	−37.04	85.15	46.67	5
DENMARK															
100%, DK	**97.9**	−83.8	396.7	−66.7	0	−233	127.3		39.7	145	61.3	122.2	88.9	173.3	125
75%, DK	**67.8**	−60.8	323.3	−66.7	0	−233	127.3		30.5	115	24.4	66.7	82.9	166.7	92.5
50%, DK	**43**	−39.5	246.7	−66.7	100	−233	127.3		20.8	80	−9.5	0	76.8	153.3	70
25%, DK	**28.7**	−19.8	160	−66.7	100	0	127.3		10.7	45	−54.5	−55.6	70.8	140	47.5
0%, DK	**2.3**	0	0	−66.7	0	100	0		0	10	−93.6	−111.1	64.8	133.3	25
Criteria weights															
WEIGHTS		0.0605	0.0968	0.0403	0.0081	0.0524	0.0282	0.0202	0.121	0.0766	0.1129	0.125	0.1048	0.0685	0.0847

The partial values presented in Table 4 are the result of transforming performance (i.e., the evidence) into value as judged by the experts, reflecting how much the experts valued different changes in performance (increase or decrease) in each criterion. All value scales for the model can be found in S5 Appendix. For some criteria, the value scales were linear (positive or negative), i.e., an increase of one unit in performance level corresponded to an increase of one unit in value. For example, the value function associated with the “diet-related health outcomes” criterion was negative linear, meaning that a decrease of one DALY was valued one point. This indicates that the experts considered each year of life saved equally valuable. Consequently, the prevention of 21 years of life lost (−20.8 DALYs) resulting from the scenario “50% substitution of beef by pulses” in Denmark (see S3 Appendix), compared to the prevention of 11 DALYs in the scenario “25% substitution”, was considered twice as valuable (20.8 vs 10.7, as shown in Tables 2 and 3). For other criteria, the relationship was not linear. For example, for the criterion “climate change”, the experts valued the decrease in GHG emissions from 0.9 kg CO2eq (“25% substitution” scenario in Portugal) to 0.6 kg CO2eq (“50% substitution) less highly (0 vs 66.67) than the decrease from 0.4 to 0.1 kg CO2eq in the “75%” and “100%” substitution scenarios, which had partial scores of 111.11 and 133.33, respectively, as shown in Tables 2 and 3 (see S4 Appendix for performance values).

The global scores of the alternatives were obtained using the aggregation model defined in Eq. (2), which combines the partial values of each criterion with the weights elicited from the expert panel.

### 3.3. Model testing and validation

**Fig 4:**
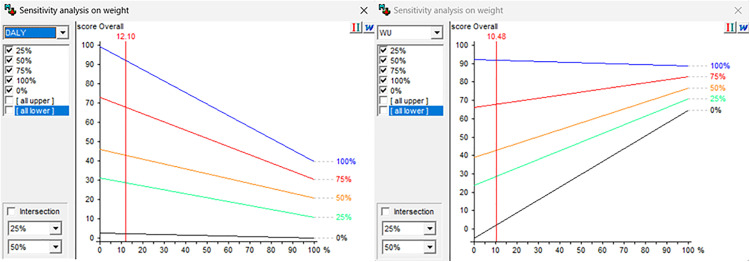
Results of the sensitivity analysis for the “diet-related health outcomes” (A) and “water use” (B) criteria of the evaluation of five alternatives of food substitutions in the diet in the Danish case study.

The sensitivity analyses conducted to understand the extent to which changes to the criteria weights would change the overall scores of the alternatives based on the experts’ feedback showed that no variation in weights would be significant enough to alter the model’s recommendation. Fig 4 presents the analysis on criteria “diet-related health impacts” (A, left) and “water use” (B, right) for Denmark as examples. In both cases, results showed that the model is stable and not sensitive to weight variations. The experts reported that the overall scores aligned with their expectations and the literature.

The robustness analysis conducted to assess the impact of the uncertainty associated with making qualitative judgements and with some of the data available was considered for the value scale of”diet-related health impacts”, due to the inherent margin of error in the source data;”environment-related health impacts”, due to sparsity of data for the Portuguese case study; and”acceptance”, because data from population surveys were not available. In all three cases, the matrix remained unchanged, showing that the 100% substitution scenario remained the best alternative with all the added uncertainty (see S6 Appendix).

## 4. Discussion

This study demonstrated how a structured socio-technical approach assisted a multidisciplinary group of experts in evaluating scenarios for dietary shifts and generating estimates of the integrated impacts of different scenarios of food substitutions. To the best of our knowledge, we present the first attempt to develop a re-useable multi-criteria model to estimate the integrated impacts of food substitutions, accounting for health, environmental, social and economic impacts of different alternatives. Unlike previous studies that assessed dietary substitutions within single domains (e.g., health or environment) or relied on simplified ordinal rankings, our study develops a reusable multicriteria model that integrates health, environmental, economic, and social dimensions in a single value framework. By combining structured expert elicitation with evidence synthesis and applying a value-measurement approach (MACBETH), this work provides, to our knowledge, the first attempt to generate a transparent, adaptable, and policy-relevant tool for assessing the integrated impacts of dietary shifts. The approach used data and evidence collection to inform multicriteria modelling, and the model was developed iteratively, with expert input and validation at each step, ensuring transparency and increasing trust in its use. This participatory process was critical to define the evaluation criteria, synthesize evidence, and elicit preferences in a transparent and reproducible manner. The approach successfully evaluated scenarios of dietary transitions in the two different populations by gathering the input of experts in four different fields (i.e., health, environment, economics and social implications of food systems), as well as available evidence on the individual dimensions. It tackled the complex challenge of accounting for diverse metrics and indicators, and different values that are inherent to evaluations of food systems and the multi-actor decision-making that is required to drive transformations of food consumption and productions towards improved health and sustainability.

### 4.1. Overall impact of alternatives of substitution of beef by pulses

In our case-study, the integrated impact of all alternatives demonstrated an overall positive impact of the substitution of consumption of beef by pulses. Results showed that the higher the proportion of substitution of consumption of beef by pulses in the population, the higher the overall benefit. This applied to the two countries, albeit with differences in the overall evaluation. These results suggest that the impact of the increasing substitution amounts of this animal-source protein (beef) by a plant-based alternative (pulses) is largely positive and reflect benefits in several criteria.

Not surprisingly, the overall positive impact was largely driven by beneficial impacts on human health and on the environment. Consumption of red meat is associated with higher risk of colon cancer, colorectal cancer, and type II diabetes [58,59]. In contrast, intake of pulses such beans, lentils, chickpeas, and others, is associated with health benefits by contributing to the prevention of ischemic heart disease [60]. The low consumption of pulses has been ranked as an important risk factor for burden of disease in many parts of the world, and, according to the Global Burden of Disease study, caused over 697,000 DALYs and 40,474 deaths in 2021 in the European region [61]. Even in regions where several types of pulses have been an integral part of diets, like the Mediterranean, their consumption [and consequently production] has decreased over the years [62–64]. Our results support that pulses are a healthy and environmentally friendly plant-based alternative source of protein, and that the transition is beneficial in all alternatives of substitution amounts when accounting for all trade-offs. It is noteworthy that the evidence we gathered was not comprehensive and did not consider, e.g., potential health outcomes associated with exposure to chemical contaminants in the foods, or with other nutrients (e.g., iron).

Previous studies have assessed the individual health and environmental impacts of these two food systems in different contexts, and some have investigated similar food substitutions (e.g., [54,65,66]). Our study leveraged on the evidence generated by these studies, as well as other data, to reach an overall integrated assessment. This methodology will be useful to assess the impact of other possible dietary shifts in populations.

Our results are in line with the conclusions of other studies that investigated individually the health, environmental, or socio-economic impacts of decreasing meat consumption and replacing with alternative protein sources in the diet individually (e.g., Cellura et al. (2022), Gazan et al., Fabricius et al.). To these methods and results, our study adds the integration of all criteria within these dimensions that experts identified as relevant, and their participation in valuing their importance for a weighted assessment.

### 4.2. Potential for use to assess other dietary shifts

MCDA has been used to address complex questions related to the impacts of interventions in the field of agriculture [67], risk ranking in food safety [68] and has been described as a useful method for risk-benefit assessment of foods [69,70]. To the best of our knowledge, this study presents the first multicriteria model developed specifically for this purpose. The study adhered to a robust multi-criteria conceptual framework, MACBETH, ensuring both axiomatic rigor and appropriate procedures for preference modelling. It followed established best practices of multi-criteria value assessment [33,34,71], which, as experts in the field have observed, are not commonly implemented when MCDA is initially applied in a new domain [72]. Therefore, this study marks a significant advancement by incorporating these standards from the outset.

The developed multicriteria model to assess the impact of dietary shifts exhibited both flexibility and adaptability: it can handle contexts with limited scientific evidence, as is often the case for some criteria in different countries, and it is adaptable for future evaluations of other dietary alternatives. The scenarios assessed were defined to represent simplified possible changes of consumption of the two foods at the population level, which, while simplified, are useful to inform policies such as national dietary guidelines. Although the model was developed within the specific context of a case study analysing the substitution of beef with pulses in Portugal and Denmark, the stakeholders aimed for it to be generalizable. This means that some criteria may not be directly applicable to the specific case study but could prove relevant in other contexts. For instance, the accessibility of the two foods considered in the case study was not a large issue in Portugal and Denmark, where imports and exports of food products are common, ensuring a consistent supply of diverse food options. However, if applied to a developing country or to substitution scenarios including novel foods in the EU (e.g., insects), accessibility could become a crucial concern. Furthermore, descriptors of performance were developed for each of the 14 evaluation criteria, making the multicriteria evaluation model independent of the four alternatives under analysis. This also ensured that the model can be reused in the future for assessing other types of dietary alternatives. Hence, our model filled a need to holistic assessments of food systems that is essential to evaluate options for policies that transcend disciplinary silos and contribute to transformation of food systems towards sustainability and health [8,73]. When applied to other case studies, our model could be able to identify which alternative scenario would have a better integrated impact when accounting for all trade-offs. For example, it is possible that a 50% substitution would have a better impact than 75%, reflecting an important negative impact for health (for example due to insufficient intake of some nutrients); for economic indicators (for example due to large negative impacts on profitability, or substitution by foods that are less affordable), or others. In general, the integrated assessment approach that we propose is able to account for trade-offs as well as identify potential breakpoints from alternatives that are beneficial to alternatives that have negative impacts. While some adaptation may be useful in very different contexts, the model developed here is reusable and adaptable in practice, providing a robust basis for assessing similar dietary substitutions across European food systems.

### 4.3. Perspectives

A limitation of this study is the construction of the model based on input from a limited number of experts. This may not adequately capture the full spectrum of perspectives and expertise required for a comprehensive evaluation. To address this, future research could employ participatory approaches such as the Delphi method, which facilitates the collection of a wider range of expert opinions through iterative rounds of feedback and consensus-building. This approach would enhance the robustness and validity of the model parameters, ensuring they more accurately reflect the complexity and variability inherent in the evaluation of dietary alternatives across different countries and dietary alternatives.

The experts identified a wide range of criteria relevant within the four dimensions. Of all criteria identified, we were able to include all except “animal welfare” (see S1 Appendix). None of the experts had the necessary expertise in animal welfare, and thus an external expert was brought on. After receiving the needed information about the study’s aims and process and given proper contextualization, the expert determined that the definition of the substitution scenarios did not consider whether the animal products in question originated from intensive or non-intensive production practices, which is important to assess the level of animal welfare of a food system. It was therefore concluded that this could not be considered a key concern in this model and the criterion was removed. Future studies should aim for structuring the problem and collecting evidence in a way that allows for the inclusion of animal welfare considerations.

In the model structuring phase of this study, we conducted an evidence synthesis that strived to collect the most representative and updated data on the two food systems for the two countries. Still, we faced data gaps that led to uncertainty in the estimates. As an ever-increasing amount and variety of data becomes available, future studies will be able to reduce these uncertainties and address similar complex questions in other countries. For example, the protein content and quality of the two different foods were not considered in the health impact evidence collected in our study. Future studies could consider an accurate evaluation of protein quality. A further limitation of our work is that the expert panel was restricted to academic experts. While this was appropriate for the methodological development and testing of the multicriteria model, future applications should expand to include a broader set of stakeholders across the food system — such as consumers, producers, industry representatives, and policymakers — to enhance legitimacy and capture the full complexity of decision-making contexts.

## 5. Conclusion

The multicriteria evaluation model we propose, combined with available evidence on the health, environmental, economic and social impacts of food systems, is useful to assess the integrated impacts of dietary shifts in specific populations. Our results showed that replacement of consumption of beef by the protein-rich plant food group pulses is beneficial in all amounts in the two European populations, and that the benefits increase with each increment of the substitution proportions. This model can be re-used and has potential to assess the integrated impacts of other dietary transitions in other populations.

## Supporting information

S1 Appendix Reusable multicriteria decision model structuring. (DOCX)

S2 Appendix List and description of criteria included in the reusable multicriteria decision model. (DOCX)

S3 Appendix Detailed environmental and health evidence synthesis. (DOCX)

S4 Appendix Tables of Performance. (DOCX)

S5 Appendix Intra-criteria value functions for the reusable multicriteria decision model. (DOCX)

S6 Appendix Sensitivity analysis. (DOCX)

S7 Appendix Robustness analysis. (DOCX)
