# Supplementary material for: Reusable multicriteria decision model to evaluate the integrated sustainability impacts of different alternatives of dietary substitutions
Source: PLoS One. 2026 Feb 25;21(2):e0339454. doi: 10.1371/journal.pone.0339454 (PMC12935239; doi:10.1371/journal.pone.0339454)
Supplement: S1 Appendix — (DOCX) [file pone.0339454.s001.docx]

# Appendix 1. Model structuring

Figure S1.1. presents the criteria and the nodes branching from the criteria that represent the concerns identified by the experts arranged into a value tree. The value tree and the corresponding definitions of each criterion were then distributed among the experts for reflection. During this discussion, experts decided that ”employment” should be moved into the economic dimension where it would be clustered with ”economic spill-over” in the ”local economic development” criterion. It was also concluded that it was necessary to include a criterion to represent the health impacts caused by the environmental consequences of the production processes (i.e., environment-related health impacts”), and that ”biodiversity” should be a criterion of its own.


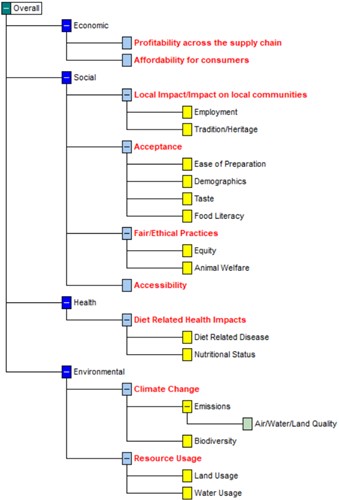


Figure S1.1. Value tree after the first brainstorming session

Following that process, an updated value tree was produced (Figure S1.2., also presented in the manuscript (Figure 3)). This value tree included the criterion “animal welfare” under the social dimension. Because none of the experts had the necessary expertise in animal welfare, an external expert was brought to support its operationalization. After receiving the needed information about the study’s aims and process and given proper contextualization, the expert determined that the definition of the substitution scenarios did not consider whether the animal products in question originated from intensive or non-intensive production practices, which is important to assess the level of animal welfare of a food system. Thus, it was concluded that this could not be considered a key concern in this model and the criterion was removed.


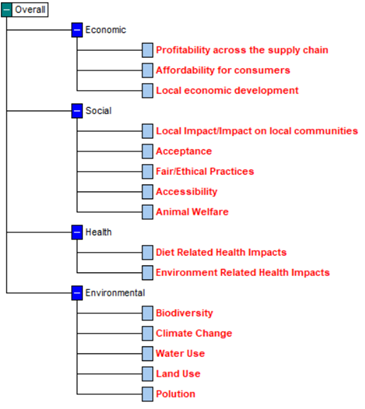


Figure S1.2. Final value tree depicting the four dimensions, with the respective criteria highlighted in bold red identified to assess the integrated health and sustainability impacts of different scenarios of dietary substitutions.  Animal welfare was selected as a criterion but not included it in the model because the definition of the substitution scenarios did not consider whether the animal products in question originated from intensive or non-intensive production practices, which is important to assess the level of animal welfare of a food system.
