# Supplementary material for: Reusable multicriteria decision model to evaluate the integrated sustainability impacts of different alternatives of dietary substitutions
Source: PLoS One. 2026 Feb 25;21(2):e0339454. doi: 10.1371/journal.pone.0339454 (PMC12935239; doi:10.1371/journal.pone.0339454)
Supplement: S2 Appendix — (DOCX) [file pone.0339454.s002.docx]

# Appendix 2. List and description of criteria included in the model

Table S2.1. List of criteria, organized by dimension, with their corresponding descriptors.

| **Criterion** | **Description** | **Descriptor** |
| --- | --- | --- |
| *Economic* | | |
| Financial performance of the farms | Considers the profitability of all production processes across the supply chain | Gross margins, revenues (%)  60%; 40% (good); 20; 0% (neutral); -20%; -40%; 60% |
| Affordability for consumers | Considers how affordable the end product is for the final consumer | Purchasing power parity (%)  60%; 30%; 10% (good); 0% (neutral); -10%; -30%; 60% |
| Local development | Considers the impacts to local economies, based on employment and economic spill-over on the territory. | Local economic development  More employment, Local multiplier >2 (good); No change to employment, Local multiplier >2; More employment, Local multiplier <2; No change to employment, Local multiplier <2 (neutral); Loss of employment, Local multiplier >2; Loss of employment, Local multiplier <2 |
| *Social* | | |
| Local impact | Considers how much traditional practices and historical heritage are impacted by the change. | Increased connection to tradition (good); No connection to tradition (neutral); Decreased connection to tradition |
| Acceptance of the change | Considers how much people are willing to change their dietary habits and if there are food-related misconceptions that may have an impact. | Willingness to change, no food misconceptions (good); Willingness to change, food misconceptions exist (neutral); No willingness to change, no food misconceptions; No willingness to change, food misconceptions exist |
| Fair/Ethical practices | Considers whether the production process considers the respect for fair and ethical practices, namely regarding gender equity. | Higher % of women employed, Higher % of women in management; Higher % of woment employed, Similar % women of in management (good); Higher % of women employed, Lower % of women in management; Similar % of women employed, Higher % of women in management; Similar % of women employed, Similar % of women in management (neutral); Similar % of women employed, Lower % of women in management; Lower % of women employed, Higher % of women in management; Lower % of women employed, Similar % of women in management; Lower % of women employed, Lower % of women in management |
| Accessibility | Considers the ease of access | More accessible (good); As accessible (neutral); Less accessible |
| *Health* | | |
| Diet and Food Related Health Impacts | Considers how the change can impact people’s health, be it through diet-related disease, food safety hazards, or nutritional status | DALY/100k inhabitants  -400; -300; -200; -100 (good);  0 (neutral), 100; 200; 300; 400 |
| Environment Related Health Impacts | Considers how the change can impact people’s health, through the environmental impacts of the production process | Photochemical smog  (gr. ethene equivalent/serving size)  0; 0.1 (good); 0.3 (neutral), 0.5; 1; 2; 3 |
| *Environment* | | |
| Biodiversity | Considers the food products´ impact on biodiversity | Nr. Species destined to extinction/Year  0 (good); 5; 15; 28 (neutral); 50; 100; 250 |
| Climate Change | Considers the food products´ production process contribution to climate change | Global Warming Score (Kg CO2 Eq/Serving Size)  0; 0.3 (Good); 0.6 (Neutral); 1.5; 4 |
| Water Use | Considers the impact the production process has on the use of water resources | Liter/Serving size  0; 5; 10 (good); 30; 50; 70; 90 (neutral); 200; 350; 560 |
| Land Use | Considers the impact the production process has on the use of land resources (arable and pasture) | Sq. Meter/Serving size  0; 1.5 (good); 3 (neutral); 5; 10; 20; 40 |
| Pollution | Considers the pollution caused by the production process | Eutrophycation(gram of phosphate/Serving size)  0; 1 (good); 5(neutral); 10; 25; 40 |
