# Supplementary material for: Reusable multicriteria decision model to evaluate the integrated sustainability impacts of different alternatives of dietary substitutions
Source: PLoS One. 2026 Feb 25;21(2):e0339454. doi: 10.1371/journal.pone.0339454 (PMC12935239; doi:10.1371/journal.pone.0339454)
Supplement: S3 Appendix — (DOCX) [file pone.0339454.s003.docx]

# Appendix 3. Detailed environmental and health evidence synthesis

Table S3.1. Environmental impacts of four scenarios of substitution of consumption of beef by pulses in Portugal and Denmark. Alternative scenario (AS) 1: 25% substitution; AS2: 50%; AS3: 75%; AS4: 100%.

|  | **Denmark** | | | | | **Portugal** | | | | |
| --- | --- | --- | --- | --- | --- | --- | --- | --- | --- | --- |
| **Indicator** | **Reference** | **AS1** | **AS2** | **AS3** | **AS4** | **Reference** | **AS1** | **AS2** | **AS3** | **AS4** |
| GHG emissions (kg CO_2_eq) | 1.2 | 0.9 | 0.6 | 0.4 | 0.1 | 0.8 | 0.6 | 0.4 | 0.2 | 0 |
| Eutrophication (g PO_4_^3-^ eq) | 4 | 3.1 | 2.2 | 1.3 | 0.5 | 4.5 | 3.5 | 2.4 | 1.4 | 0.3 |
| Water Use (L) | 33.5 | 29.5 | 25.5 | 21.4 | 17.4 | 19.9 | 17.8 | 15.8 | 13.7 | 11.7 |
| Arable Land Use (m^2^*year) | 0.8 | 0.7 | 0.6 | 0.5 | 0.4 | 0.4 | 0.4 | 0.3 | 0.3 | 0.3 |
| Pasture Land Use (m^2^*year) | 0.3 | 0.2 | 0.1 | 0.1 | 0 | 1.9 | 1.4 | 0.9 | 0.5 | 0 |
| Total Land Use (m^2^*year) | 1 | 0.9 | 0.7 | 0.5 | 0.4 | 2.3 | 1.8 | 1.3 | 0.8 | 0.3 |
| Biodiversity impact | 54.4 | 42.4 | 30.5 | 18.5 | 6.6 | 28.6 | 22.6 | 16.5 | 10.5 | 4.4 |

**Table S3.2.** Estimated health impact of four scenarios of substitution of consumption of beef by pulses in Portugal and Denmark. Health impacts are associated with dietary risk factors, measured in change in years lived with disability (YLD), years of life lost YLL) and disability adjusted life years (DALY) change from current consumption in the population to each scenario (mean delta DALY and 95% Uncertainty Interval). Alternative scenario (AS) 1: 25% substitution; AS2: 50%; AS3: 75%; AS4: 100%.

|  |  | **YLD** | | | **YLL** | | | **DALY** | | |
| --- | --- | --- | --- | --- | --- | --- | --- | --- | --- | --- |
|  | **Health outcome** | **Mean** | **95% UI** | | **Mean** | **95% UI** |  | **Mean** | **95% UI** | |
| **Portugal*** | | | | | | | | | | |
| AS1 | CRC | -0.1 | 0.0 | -0.9 | -1.5 | 0.0 | -14.9 | -1.6 | 0.0 | -15.8 |
|  | Diabetes | -0.3 | 0.0 | -2.5 | -0.1 | 0.0 | -0.7 | -0.4 | 0.0 | -3.2 |
|  | IHD | -0.2 | 0.0 | -1.6 | -3.7 | 0.0 | -35.0 | -3.8 | 0.0 | -36.5 |
|  | TOTAL | -0.5 | 0.0 | -5.0 | -5.3 | 0.0 | -50.6 | -5.9 | 0.0 | -55.5 |
| AS2 | CRC | -0.2 | 0.0 | -1.7 | -3.0 | 0.0 | -29.0 | -3.2 | 0.0 | -30.7 |
|  | Diabetes | -0.5 | 0.0 | -4.7 | -0.2 | 0.0 | -1.4 | -0.7 | 0.0 | -6.1 |
|  | IHD | -0.3 | 0.0 | -3.0 | -6.8 | 0.0 | -64.6 | -7.2 | 0.0 | -67.5 |
|  | TOTAL | -1.0 | 0.0 | -9.5 | -10.0 | 0.0 | -95.0 | -11.1 | 0.0 | -104.3 |
| AS3 | CRC | -0.3 | 0.0 | -2.5 | -4.4 | 0.0 | -42.3 | -4.6 | 0.0 | -44.8 |
|  | Diabetes | -0.8 | 0.0 | -6.8 | -0.3 | 0.0 | -2.1 | -1.1 | 0.0 | -8.8 |
|  | IHD | -0.4 | 0.0 | -4.2 | -9.6 | 0.0 | -89.8 | -10.0 | 0.0 | -93.8 |
|  | TOTAL | -1.5 | 0.0 | -13.6 | -14.3 | 0.0 | -134.2 | -15.8 | 0.0 | -147.4 |
| AS4 | CRC | -0.3 | 0.0 | -3.3 | -5.7 | 0.0 | -54.9 | -6.0 | 0.0 | -58.2 |
|  | Diabetes | -1.0 | 0.0 | -8.7 | -0.4 | 0.0 | -2.7 | -1.4 | 0.0 | -11.2 |
|  | IHD | -0.6 | 0.0 | -5.3 | -12.0 | 0.0 | -111.3 | -12.6 | 0.0 | -116.2 |
|  | TOTAL | -1.9 | 0.0 | -17.3 | -18.1 | 0.0 | -168.8 | -20.0 | 0.0 | -185.6 |
| **Denmark*** | | | | | | | | | | |
| AS1 | CRC | -0.3 | 0.0 | -1.3 | -5.2 | 0.0 | -22.7 | -5.4 | 0.0 | -23.9 |
|  | Diabetes | -0.7 | 0.0 | -3.1 | -0.4 | 0.0 | -2.6 | -1.1 | 0.0 | -5.4 |
|  | IHD | -0.2 | 0.0 | -0.9 | -3.9 | 0.0 | -16.6 | -4.1 | 0.0 | -17.3 |
|  | TOTAL | -1.2 | 0.0 | -5.2 | -9.5 | 0.0 | -41.8 | -10.7 | 0.0 | -46.6 |
| AS2 | CRC | -0.6 | 0.0 | -2.5 | -10.2 | 0.0 | -44.3 | -10.7 | 0.0 | -46.7 |
|  | Diabetes | -1.3 | 0.0 | -5.9 | -0.8 | 0.0 | -5.1 | -2.1 | 0.0 | -10.6 |
|  | IHD | -0.4 | 0.0 | -1.7 | -7.5 | 0.0 | -30.9 | -7.9 | 0.0 | -32.4 |
|  | TOTAL | -2.3 | 0.0 | -10.1 | -18.5 | 0.0 | -80.3 | -20.8 | 0.0 | -89.6 |
| AS3 | CRC | -0.8 | 0.0 | -3.6 | -15.1 | 0.0 | -65.0 | -15.9 | 0.0 | -68.4 |
|  | Diabetes | -2.0 | 0.0 | -8.6 | -1.2 | 0.0 | -7.4 | -3.1 | 0.0 | -15.5 |
|  | IHD | -0.6 | 0.0 | -2.4 | -10.9 | 0.0 | -43.4 | -11.5 | 0.0 | -45.5 |
|  | TOTAL | -3.4 | 0.0 | -14.7 | -27.1 | 0.0 | -115.9 | -30.5 | 0.0 | -129.4 |
| AS4 | CRC | -1.1 | 0.0 | -4.7 | -19.8 | 0.0 | -84.8 | -20.9 | 0.0 | -89.2 |
|  | Diabetes | -2.6 | 0.0 | -11.2 | -1.5 | 0.0 | -9.7 | -4.1 | 0.0 | -20.1 |
|  | IHD | -0.8 | 0.0 | -3.0 | -13.9 | 0.0 | -54.3 | -14.7 | 0.0 | -56.8 |
|  | TOTAL | -4.5 | 0.0 | -18.9 | -35.3 | 0.0 | -148.8 | -39.7 | 0.0 | -166.2 |

CRC: colorectal cancer. IHD: ischemic heart disease. YLD: years lived with disability. YLL: years of life lost YLL. DALY: disability adjusted life years. *Estimated based on the model by Fabricius et al., 2021.
