# Supplementary material for: Reusable multicriteria decision model to evaluate the integrated sustainability impacts of different alternatives of dietary substitutions
Source: PLoS One. 2026 Feb 25;21(2):e0339454. doi: 10.1371/journal.pone.0339454 (PMC12935239; doi:10.1371/journal.pone.0339454)
Supplement: S4 Appendix — (DOCX) [file pone.0339454.s004.docx]

# Appendix 4. Tables of Performance

Table S4.1. Table of performances for 14 criteria of four scenarios of substitution of consumption of beef by pulses in Denmark. Alternative scenario (AS) 1: 25% substitution; AS2: 50%; AS3: 75%; AS4: 100%.

|  | **Scenario** | | | | |
| --- | --- | --- | --- | --- | --- |
| **Criteria** | **Reference** | **AS1. 25% Substitution** | **AS2. 50% Substitution** | **AS3. 75% Substitution** | **AS4. 100% Substitution** |
| *Economic* | | | | | |
| Profitability | 0 | -7.9 | -15.8 | -23.7 | -31.6 |
| Affordability | 0 | 22 | 44 | 67 | 89 |
| Local economic development | Level 2 | Level 6 | Level 6 | Level 6 | Level 6 |
| *Social* | | | | | |
| Local impact | Level 2 | Level 1 | Level 1 | Level 2 | Level 2 |
| Acceptance | Level 1 | Level 2 | Level 4 | Level 4 | Level 4 |
| Fair/Ethical Practices | Level 5 | Level 1 | Level 1 | Level 1 | Level 1 |
| Accessibillity | Level 2 | Level 2 | Level 2 | Level 2 | Level 2 |
| *Health* | | | | | |
| Diet-related Health Impacts | 0 | -10.7 | -20.8 | -30.5 | -39.7 |
| Environment-related Health Impacts | 0.28 | 0.21 | 0.14 | 0.07 | 0.01 |
| *Environment* | | | | | |
| Biodiversity | 54.4 | 42.4 | 30.5 | 18.5 | 6.6 |
| Climate Change | 1.2 | 0.9 | 0.6 | 0.4 | 0.1 |
| Water Use | 33.5 | 29.5 | 25.5 | 21.4 | 17.4 |
| Land Use | 1 | 0.9 | 0.6 | 0.4 | 0.1 |
| Polution | 4 | 3.1 | 2.2 | 1.3 | 0.5 |

Table S4.2. Table of performances for 14 criteria of four scenarios of substitution of consumption of beef by pulses in Portugal. Alternative scenario (AS) 1: 25% substitution; AS2: 50%; AS3: 75%; AS4: 100%.

|  | **Scenario** | | | | |
| --- | --- | --- | --- | --- | --- |
| **Criteria** | **Reference** | **AS1. 25% Substitution** | **AS2. 50% Substitution** | **AS3. 75% Substitution** | **AS4. 100% Substitution** |
| *Economic* | | | | | |
| Profitability | 0 | 13.2 | 26.4 | 39.6 | 52.8 |
| Affordability | 0 | 18 | 37 | 56 | 74 |
| Local economic development | Level 2 | Level 6 | Level 6 | Level 6 | Level 6 |
| *Social* | | | | | |
| Local impact | Level 2 | Level 1 | Level 1 | Level 2 | Level 2 |
| Acceptance | Level 1 | Level 2 | Level 4 | Level 4 | Level 4 |
| Fair/Ethical Practices | Level 5 | Level 1 | Level 1 | Level 1 | Level 1 |
| Accessibillity | Level 2 | Level 2 | Level 2 | Level 2 | Level 2 |
| *Health* | | | | | |
| Diet-related Health Impacts | 0 | -5.9 | -11.1 | -15.8 | -20 |
| Environment-related Health Impacts* | 0.28 | 0.21 | 0.14 | 0.07 | 0.01 |
| *Environment* | | | | | |
| Biodiversity | 28.6 | 22.6 | 16.5 | 10.5 | 4.4 |
| Climate Change | 0.8 | 0.6 | 0.4 | 0.2 | 0 |
| Water Use | 19.9 | 17.8 | 15.8 | 13.7 | 11.7 |
| Land Use | 2.3 | 1.8 | 1.3 | 0.8 | 0.3 |
| Polution | 4.5 | 3.5 | 2.4 | 1.4 | 0.5 |

*Surrogate date from Danish case study.
