# Supplementary material for: Reusable multicriteria decision model to evaluate the integrated sustainability impacts of different alternatives of dietary substitutions
Source: PLoS One. 2026 Feb 25;21(2):e0339454. doi: 10.1371/journal.pone.0339454 (PMC12935239; doi:10.1371/journal.pone.0339454)
Supplement: S5 Appendix — (DOCX) [file pone.0339454.s005.docx]

# Appendix 5. Intra-criteria value functions for the model

Building of value functions was performed with the same groups that performed the criteria operationalization. The basis for this stage was the anchoring of the ”neutral” and ”good” reference levels on every descriptor to value scores of ”0” and ”100”, respectively. The qualitative judgements provided by the participants built upon that to build the value scale. Participants were asked to perform pairwise comparisons and judge the differences in value between the levels of performance, filling out a matrix in the process. Figures S5.1 to S5.11 present the judgement matrixes for all the criteria, alongside their respective value functions or value scales.


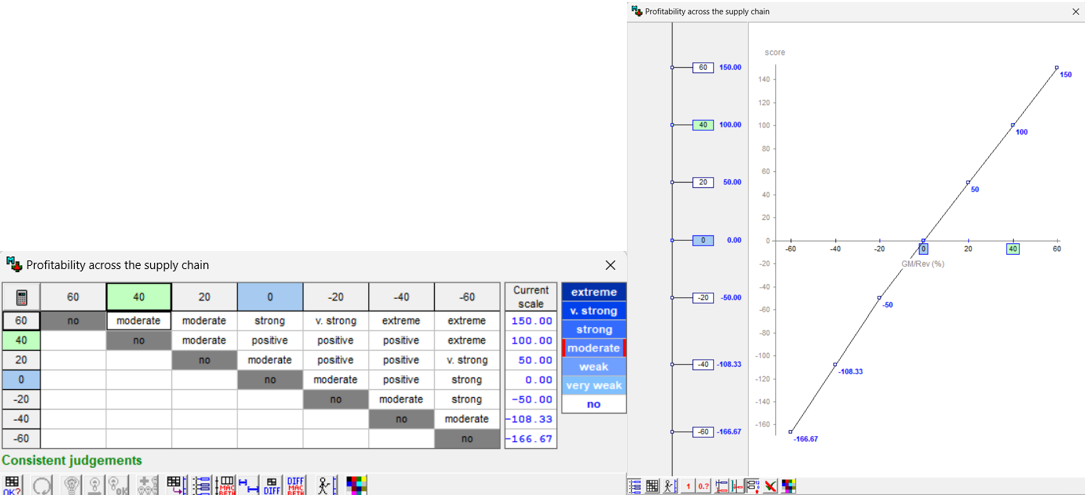
Figure S5.1. ”Profitability” judgement matrix and value function.


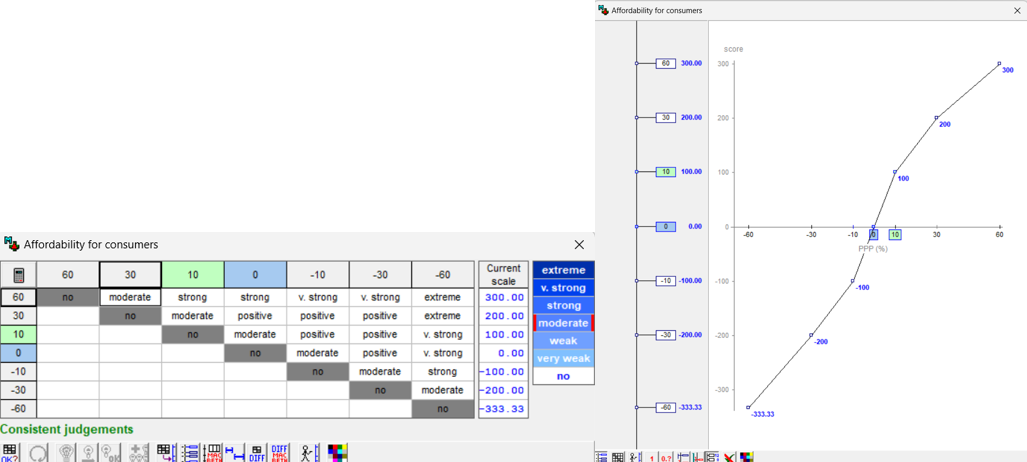


Figure S5.2. ”Affordability” judgement matrix and value function


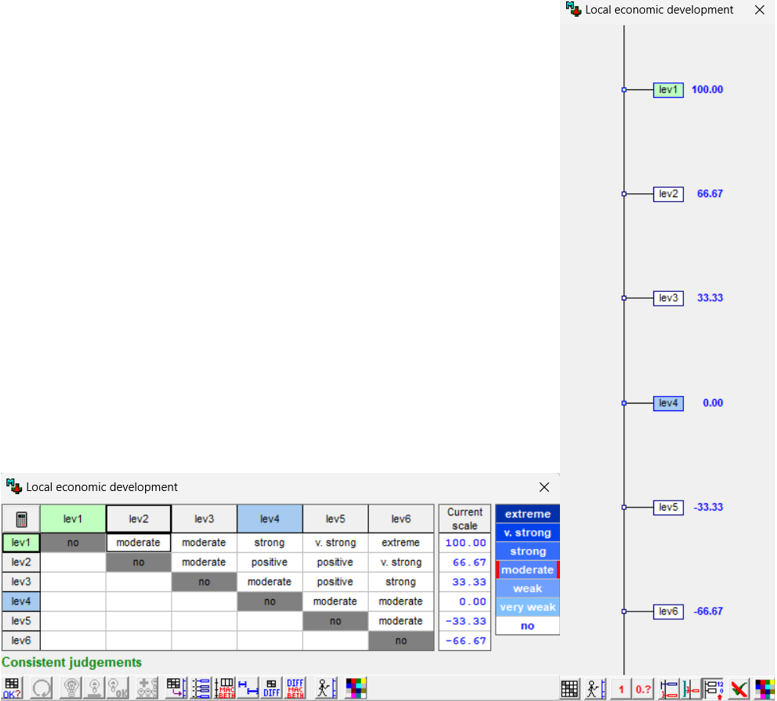


Figure S5.3. ”Local economic development” judgement matrix and value function


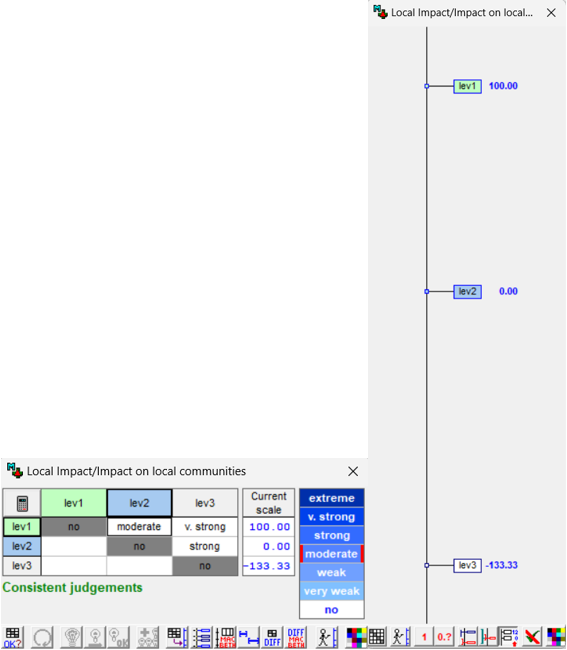


Figure S5.4. ”Local impact” judgement matrix and value function


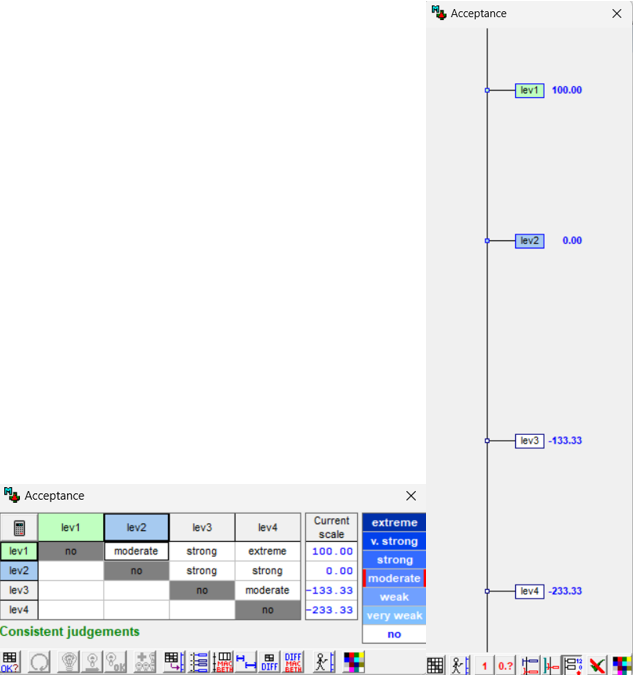


Figure S5.5. ” Acceptance” judgement matrix and value function


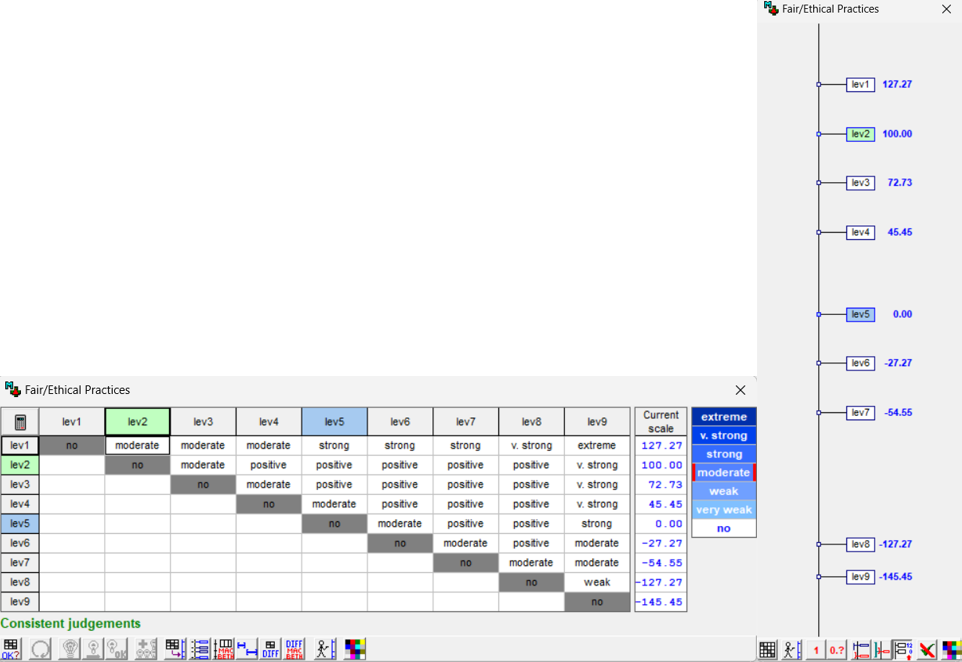


Figure S5.6. ” Fair and ethical practices” judgement matrix and value function


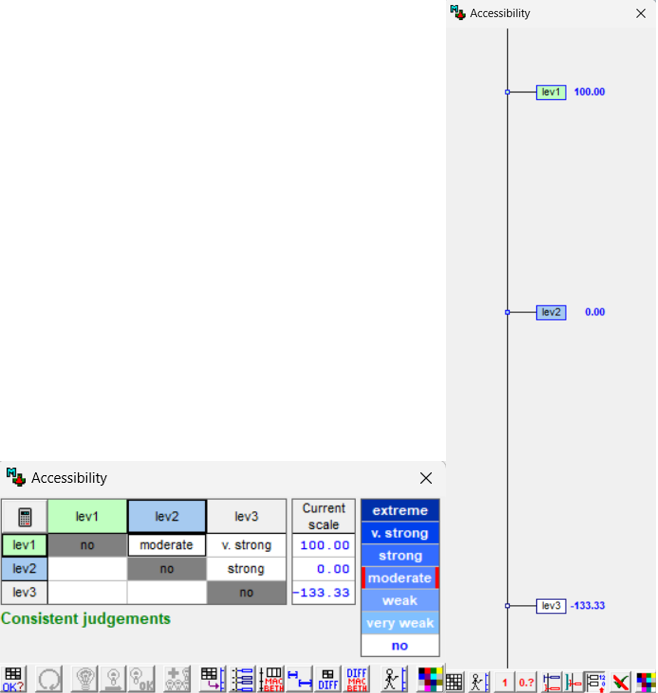


Figure S5.7. ” Accessibility” judgement matrix and value function


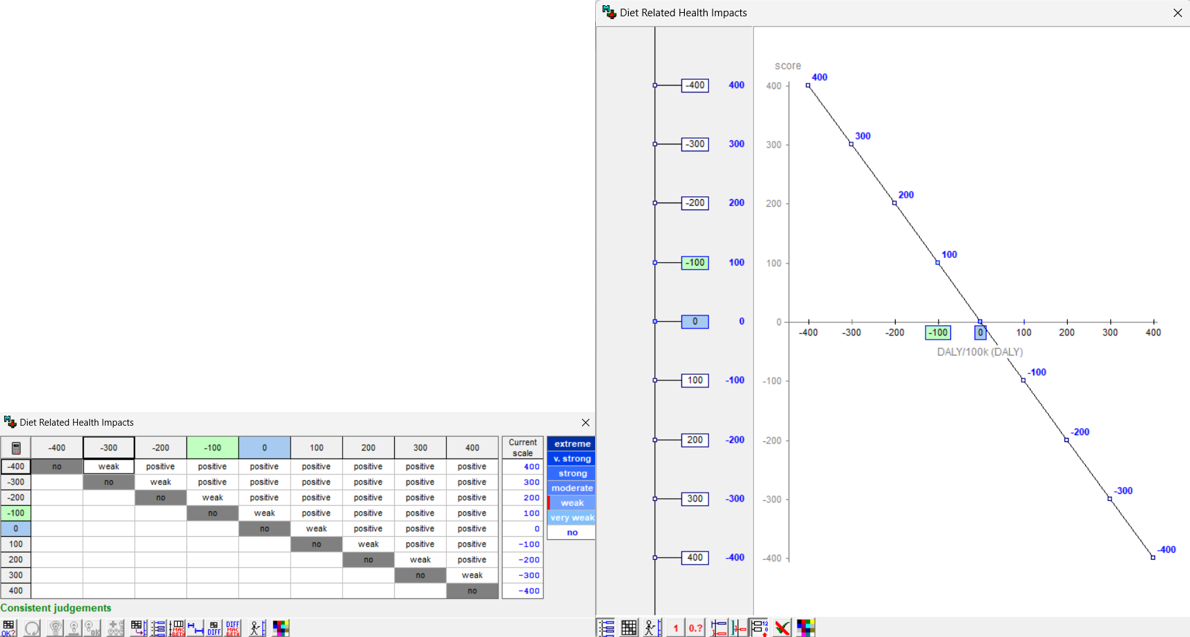


Figure S5.8. ”Diet-related health impacts” judgement matrix and value function


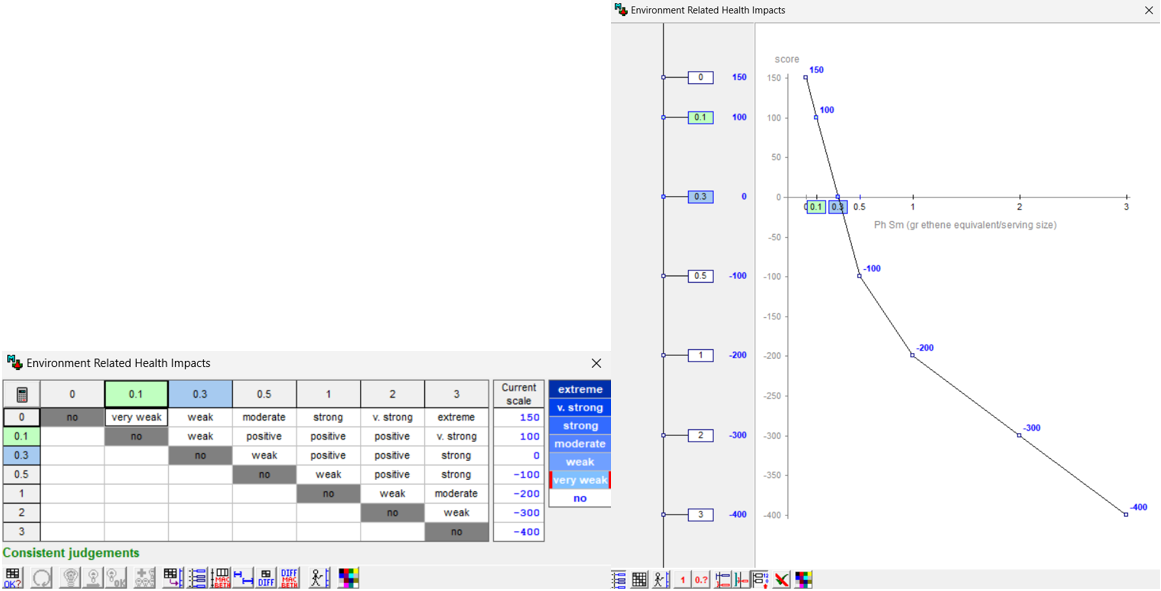


Figure S5.9. ”Environment-related health impacts” judgement matrix and value function


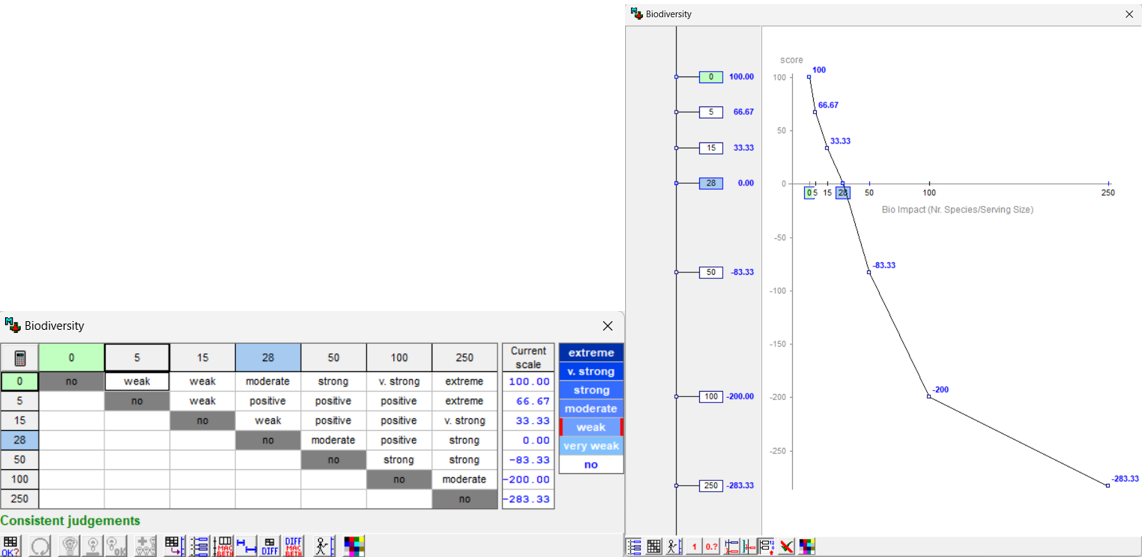


Figure S5.10. ” Biodiversity” judgement matrix and value function


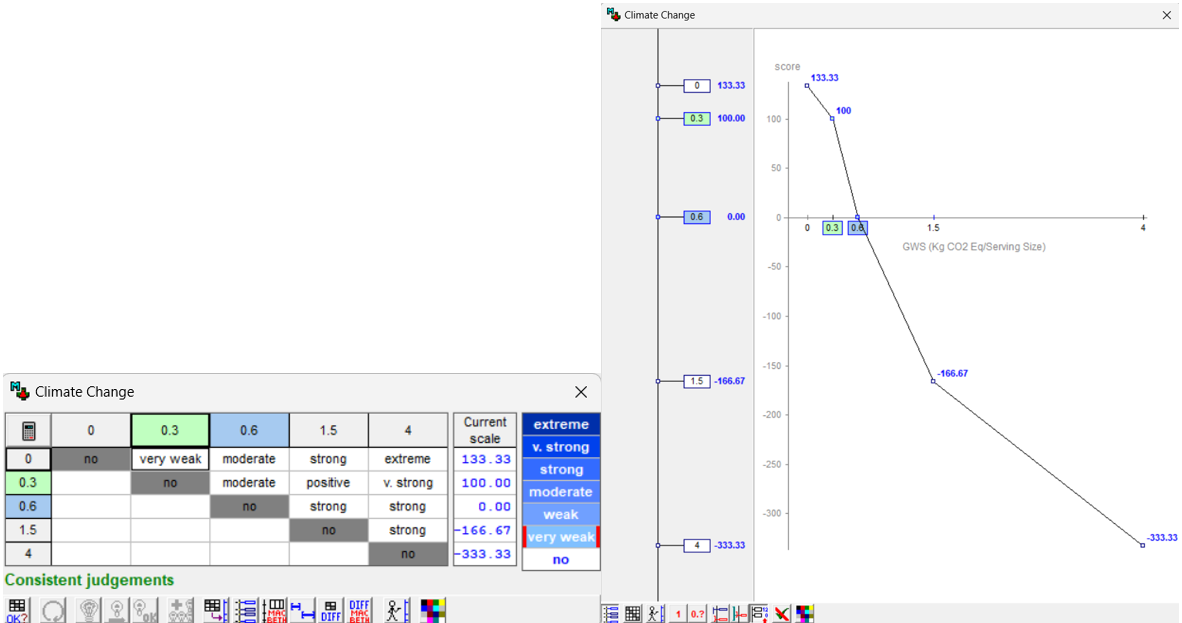


Figure S5.11. ”Climate change” judgement matrix and value function


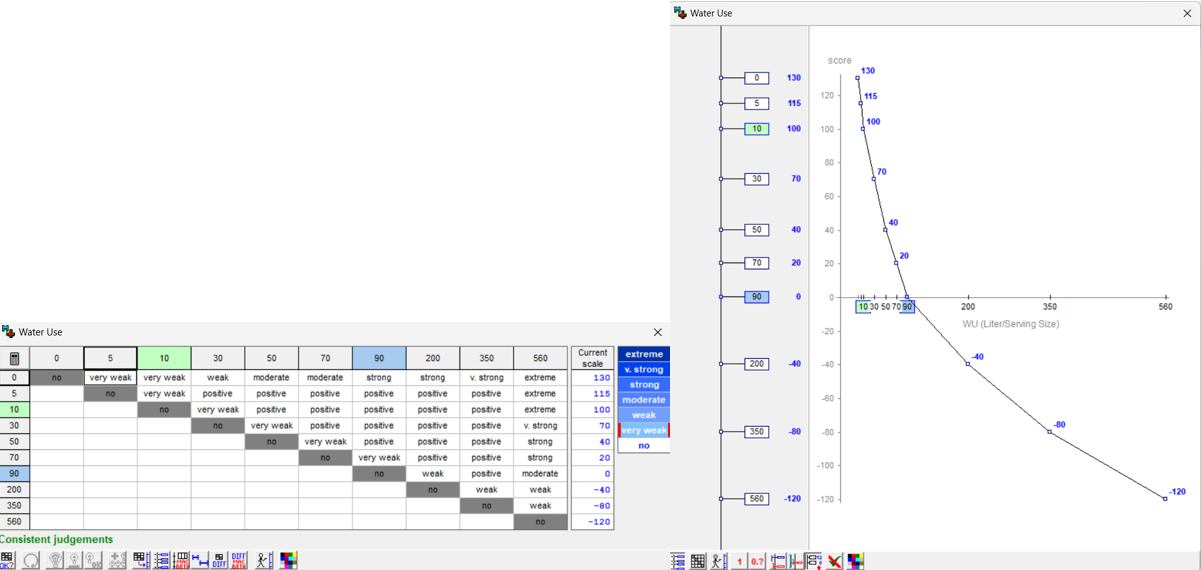


Figure S5.11. ”Water use” judgement matrix and value function


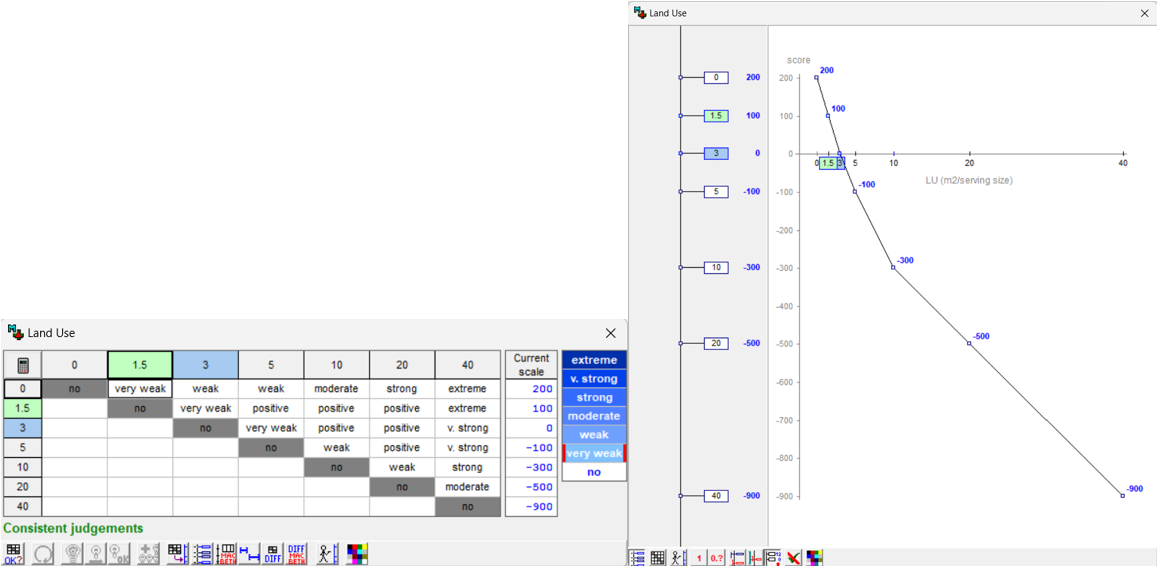


Figure S5.11. ”Land use” judgement matrix and value function


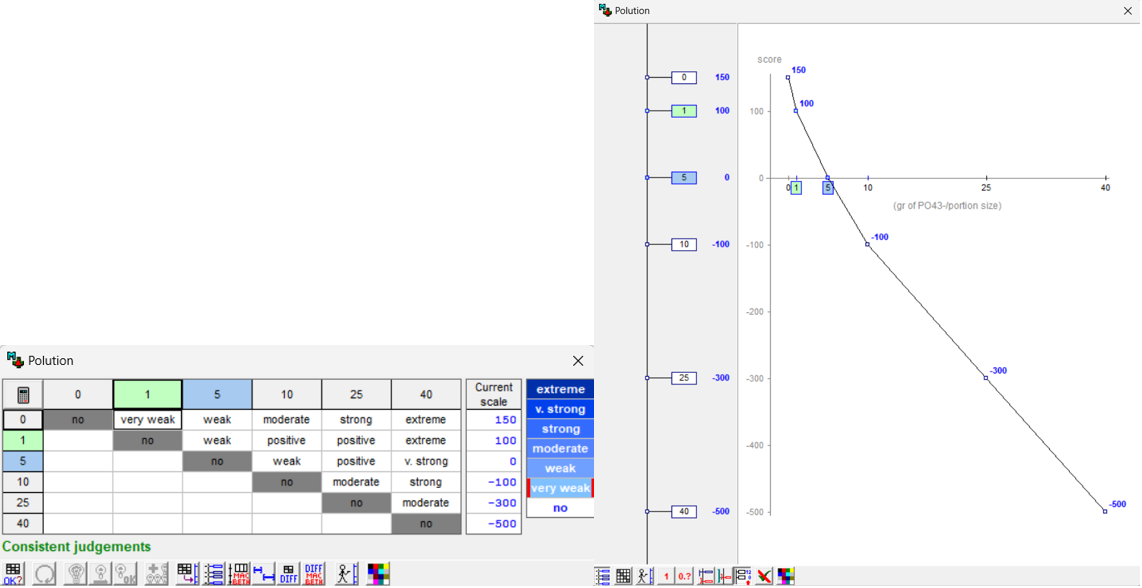


Figure S5.11. ”Pollution” judgement matrix and value function
