# Supplementary material for: Reusable multicriteria decision model to evaluate the integrated sustainability impacts of different alternatives of dietary substitutions
Source: PLoS One. 2026 Feb 25;21(2):e0339454. doi: 10.1371/journal.pone.0339454 (PMC12935239; doi:10.1371/journal.pone.0339454)
Supplement: S6 Appendix — (DOCX) [file pone.0339454.s006.docx]

# Appendix 6. Sensitivity analysis

A sensitivity analysis was conducted on the weights for every criterion. We present the graphs corresponding to the criteria ”diet-related health impacts” and ”water use” as examples (Figures S6.1 and S6.2). The figures demonstrate that no variation in the weights would be great enough to lead to changes in the model’s recommendation. For the remaining criterion, there was no case in which a reasonable variation in weights would result in changes to the recommendation. Therefore, we concluded that the model is stable and not sensitive to weight variations.


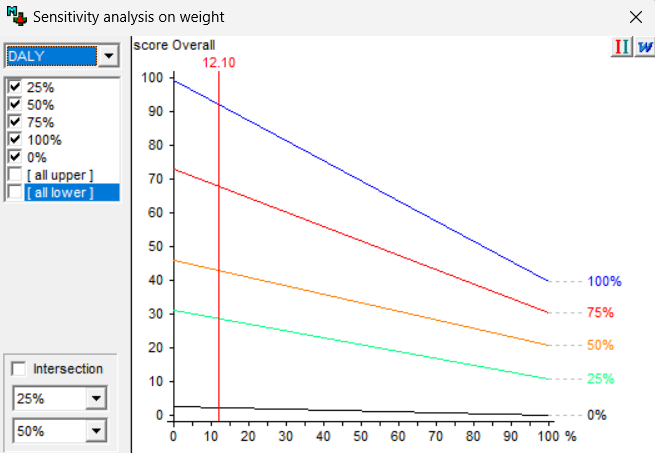


Figure S6.1: Sensitivity analysis for the ”Diet-related health impacts” criterion in the Danish case study.


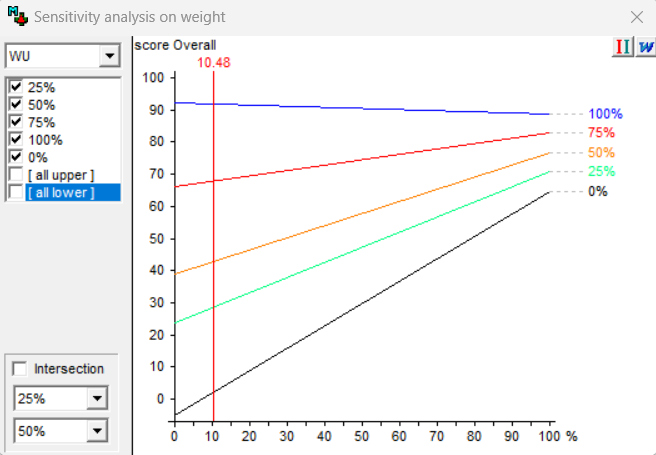


Figure S6.2: Sensitivity analysis for the ”Water use” criterion in the Danish case study.
