# Supplementary material for: Reusable multicriteria decision model to evaluate the integrated sustainability impacts of different alternatives of dietary substitutions
Source: PLoS One. 2026 Feb 25;21(2):e0339454. doi: 10.1371/journal.pone.0339454 (PMC12935239; doi:10.1371/journal.pone.0339454)
Supplement: S7 Appendix — (DOCX) [file pone.0339454.s007.docx]

# Appendix 7. Robustness analysis

Due to the uncertainty that can be associated with making qualitative judgements and that comes with the data itself, it was necessary to validate the robustness of the model and its conclusions. The M-MACBETH robustness analysis starts with a matrix comprising all the scenarios and filled out by symbols that represent different types of dominance: red triangles for classic dominance, where one option dominates another if it is more preferred in at least one criterion and not less preferred in any other; green plus signs for additive dominance, where one option dominates another if it is always more attractive through the use of an additive model under a set of constraints. If none of those two situations apply, a question mark is displayed. The software organizes the information it receives into two sections (local and global) and three 62 types (ordinal, MACBETH and cardinal). Local information refers to information specific to a criterion, while global information is relative to the weights. Ordinal information pertains only to the ranking of the scenarios, not considering judgements regarding differences of attractiveness. Those are considered under the MACBETH information type. Cardinal information refers to the specific scales validated by the decision maker. Considering the number of close votes during the weighting process, uncertainty was added to the weights in figure S7.1. As a result, the ranking of the scenarios did not change, although there is no longer dominance between ”All upper” and ”100%”, nor between ”0%” and ”All lower”. Uncertainty was also introduced into the value functions of ”diet-related health impacts”, due to the inherent margin of error in the source data, ”environment-related health impacts”, due to data availability issues for the Portuguese case study, and ”acceptance”, due to the estimates being made in the place of population surveys. In all three cases, the matrix remained unchanged. We therefore concluded that the 100% substitution scenario is a robust one since it remains the best with all the added uncertainty.


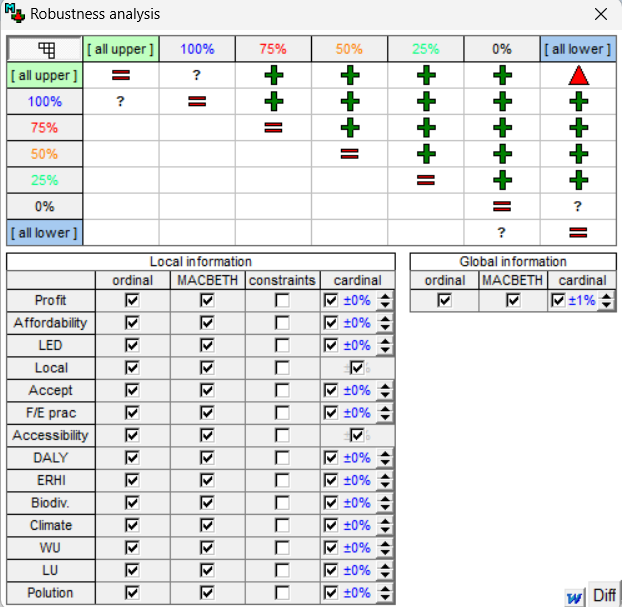


Figure S7.1. Robustness analysis for weight uncertainty in the Danish case study
